# Effects of methionine supplementation in a reduced protein diet on growth performance, oxidative status, intestinal health, oocyst shedding, and methionine and folate metabolism in broilers under *Eimeria* challenge

**DOI:** 10.1186/s40104-024-01041-4

**Published:** 2024-06-10

**Authors:** Guanchen Liu, Venkata Sesha Reddy Choppa, Milan Kumar Sharma, Hanseo Ko, Janghan Choi, Woo Kyun Kim

**Affiliations:** Department of Poultry Science, University of Georgia, Athens, GA 30602 USA

**Keywords:** Broiler, Coccidiosis, *Eimeria*, Intestinal health, Methionine

## Abstract

**Background:**

This study investigated effects of different methionine (Met) supplementation levels in a reduced protein diet on growth performance, intestinal health, and different physiological parameters in broilers under *Eimeria* challenge. A total of 600 fourteen-day-old Cobb500 male broilers were challenged with *E. maxima, E. tenella,* and *E. acervulina*, and randomly allocated in a 2 × 5 factorial arrangement. Birds received normal protein diets (20% crude protein, NCP) or reduced protein diets (17% crude protein, LCP), containing 2.8, 4.4, 6.0, 7.6, and 9.2 g/kg of Met.

**Results:**

On 6 and 9 days post inoculation (DPI), increasing Met level linearly improved the growth performance (*P* < 0.05). Total oocyst shedding linearly increased as Met level increased (*P* < 0.05). Duodenal villus height (VH):crypt depth (CD) in the LCP groups were higher on 6 DPI (*P* < 0.01) while lower on 9 DPI (*P* < 0.05) compared to the NCP groups. Jejunal CD and duodenal VH:CD changed quadratically as Met level increased (*P* < 0.05). On 6 DPI, liver glutathione (GSH) and glutathione disulfide (GSSG) linearly increased as Met level increased (*P* < 0.05). On 9 DPI, GSSG quadratically increased, whereas GSH:GSSG quadratically decreased as Met levels increased (*P* < 0.05). The expression of amino acid transporters linearly decreased as Met level increased (*P* < 0.05). The expression of zonula occludens 2 and claudin-1 linearly increased on 6 DPI whereas decreased on 9 DPI as Met level increased (*P* < 0.05). The expressions of cytokines were lower in the LCP groups than the NCP groups (*P* < 0.05). Interaction effects were found for the expression of *IL-10* and *TNFα* on 6 DPI (*P* < 0.05), where it only changed quadratically in the NCP group as Met level increased. The expression of Met and folate metabolism genes were lower in the LCP groups than the NCP groups on 9 DPI (*P* < 0.05). The expression of these genes linearly or quadratically decreased as Met level increased (*P* < 0.05).

**Conclusion:**

These results revealed the regulatory roles of Met in different physiological parameters including oxidative status, intestinal health, and nutrient metabolism in birds fed reduced protein diet and challenged with *Eimeria*.

## Background

Coccidiosis is a widespread and economically significant parasitic disease affecting the poultry industry [1]. It is caused by the protozoan parasite of the genus *Eimeria*, which inflicts damage to the intestinal lining of the birds during its reproduction cycle [2]. The infection leads to compromised intestinal integrity, hindered nutrient absorption, inflammation, and oxidative stress, ultimately resulting in a substantial decline in growth performance and even mortality [3–5, 6]. Anticoccidial drugs have traditionally served as an effective method to combat coccidiosis [2]. However, concerns have been raised due to the development of drug resistance [7] and growing public apprehension regarding antibiotic use in animal production [8]. While vaccinating birds against coccidiosis has demonstrated effectiveness, achieving successful vaccination requires exposing birds to live or attenuated oocysts to develop immunity [9], which can still lead to intestinal lesions that might potentially predispose another intestinal disease necrotic enteritis [10]. With both approaches facing limitations, the exploration of alternative strategies to mitigate the impact of coccidiosis has become necessary.

Since optimal nutrient compositions in diets are crucial for sustaining the well-being of infected birds and the altered physiological state and heightened immune responses induced by coccidiosis may lead to changes in the nutrient requirements which were proposed for the healthy broilers [11, 12]. One promising alternative avenue to combat coccidiosis emerged as the nutritional interventions by increasing the supplementation of nutrients possessing function roles like amino acids into broiler diets, aiming to reduce intestinal damage and promote recovery [13]. Methionine (Met) as one of the essential amino acids is also considered the first limiting amino acid in poultry production [14, 15]. It not only plays a crucial role in maintaining bird growth and protein synthesis, but also holds significant importance in supporting the intestinal health, immune responses, and anti-oxidative functions of the birds [16–21]. Besides the established significance in protein synthesis, Met also plays a crucial role in regulating several signaling pathways, including the mTOR and Wnt/β-catenin pathways [22, 23]. These pathways are known for their role in maintaining intestinal structure by regulating the renewal and differentiation of intestinal stem cells [24]. This dual role of Met may contribute significantly to the regenerative capacity of the intestine, especially during coccidiosis infection when the intestinal epithelial cells as well as the tight junctions between them are severely damaged [5, 6]. Moreover, research has shown that Met possesses potent antioxidant capacity, attributed to its close metabolic relationship with glutathione (GSH) and its capability to scavenge free radicals [25–29]. Additionally, the critical role Met plays in the immune responses cannot be ignored, research showed that the Met as well as its metabolites *S*-adenosylmethionine (SAM) are important for the activation and proliferation of T cells [30, 31], which are essential in combating the *Eimeria* infections [32]. Sufficient supplementation of Met has also been shown to enhance antibody production in broilers [33–35]. Given the multiple functional roles Met possesses, supplementation of Met in diets of broilers under *Eimeria* challenge might potentially alleviate the impact of the infection and improve the performance of the birds.

However, the potential negative impacts of excessive Met supplementation should not be overlooked. Toxicity caused by excessive Met supplementation has been well documented [36]. While the demands for Met may increase in birds challenged by *Eimeria* due to its beneficial functional roles, over supplementation of Met could still lead to impaired growth performance. This negative effects could be intensified when the birds are under coccidiosis infection. Moreover, it’s crucial to acknowledge the intimate connection between Met and folate metabolism within the one-carbon cycle. In the process of homocysteine remethylation, elevated Met levels might contribute to the synthesis of additional tetrahydrofolate, ultimately converted into pyrimidine for DNA synthesis and cellular proliferation [37, 38]. This metabolic interplay suggests that an excess of Met might potentially heighten folate and pyrimidine availability to *Eimeria*, which are essential for their reproduction [39, 40], thus potentially exacerbating the severity of the infection by favoring the reproduction of the parasites.

Reducing the dietary protein levels in animal diets has been proposed by researchers and producers due to its various benefits [41]. One major advantage is the potential to lower production costs by decreasing the usage of high-quality protein ingredients. [13, 42]. Additionally, this approach offers environmental advantages by decreasing nitrogen excretion and ammonia emissions [41–43]. However, with the protein content reduced in the diets, it is of more importance to meet the optimal amino acid requirements to maintain the growth performance of the birds especially when the birds are under disease conditions [44, 45].

Hence, an understanding of the optimal Met supplementation levels becomes crucial for coccidia-infected birds fed a reduced protein diet, which ensures not only the economic efficiency and environmental sustainability of poultry production but also the well-being of the birds in the face of coccidiosis challenges. Despite the significance of this issue, there is a scarcity of studies addressing this topic. Therefore, the present study aims to bridge this gap by investigating the effects of different levels of Met supplementation in a reduced protein diet on the growth performance, intestinal health, immune responses, oocyst shedding, and metabolism of Met and folate in broilers challenged with *Eimeria*. Our hypothesis is that increasing Met levels could enhance the performance of broilers under *Eimeria* challenge by improving the oxidative status and intestinal health of the birds. However, we postulate that an optimal level of dietary Met may exist, beyond which further supplementation could potentially favor the reproduction of parasites and result in adverse effects.

### Materials and methods

All the animal experiment procedures used in this study were approved by the Institutional Animal Care and Use Committee of the University of Georgia (A2021 12–012).

### Birds, diets, and *Eimeria* challenge

A total of 600 one-day-old Cobb500 male broiler chicks were fed a same starter diet that met the breeder’s nutrient recommendations [46] from d 0 to 14. On d 14, all birds were orally gavaged with 1 mL of solution containing 25,000 oocysts of *E. maxima*, 25,000 oocysts of *E. tenella*, and 125,000 oocysts of *E. acervulina*. The *Eimeria* spp. utilized in this study were isolates from North Carolina field strains. The oocysts were sporulated in 2% potassium dichromate at 30 °C. Following sporulation, the oocysts were washed with PBS and quantified using a McMaster chamber (Jorgensen Laboratories, Loveland, CO, USA). Subsequently, the quantified oocysts were combined and resuspended in water to achieve the desired concentration. The birds were then randomly allocated into 10 treatments in a 2 × 5 factorial arrangement with 2 levels of crude protein and 5 levels of Met. Each treatment contained 5 replicates with 12 birds per replicate. The treatment grower diets were corn and soybean meal based and included two protein levels. The normal protein diet (NCP) contained 20% crude protein (CP) with amino acid levels, except Met, meeting the breeder’s recommendations. The reduced protein diet (LCP) contained 17% CP with amino acid levels, except Met, reduced by 15% compared to the NCP diet to achieve the similar amino acid to lysine ratios. In the NCP diet, one group received no crystalline form of DL-Met supplementation, with a Met content of 2.8 g/kg. The subsequent groups contained 1.6 g/kg more Met than the previous group, with the 6.0 g/kg Met group representing the recommended Met level by the breeders and the 9.2 g/kg Met group containing around 50% more Met than the breeder’s recommendation. In the LCP diets, DL-Met supplementation was adjusted to achieve equivalent Met levels as in the NCP diets. The corresponding diets were denoted as Met 2.8, Met 4.4, Met 6.0, Met 7.6 and Met 9.2, respectively. The diet samples were sent for CP and amino acid analysis at a commercial laboratory (The University of Missouri-Columbia Agricultural Experiment Station and Chemical Laboratories, Columbia, MO, USA). The feedstuffs and chemical composition of the diets are shown in Table 1. The birds were raised in battery cages for the entire duration of the experiment with ad libitum access to feed and water. Temperature and lighting programs followed the Cobb500 Broiler Management Guide [47].

**Table 1:** Ingredient formulation and nutrient and energy composition of experimental diets^a^

Item	NCP					LCP				
	Met 2.8	Met 4.4	Met 6.0	Met 7.6	Met 9.2	Met 2.8	Met 4.4	Met 6.0	Met 7.6	Met 9.2
Ingredient, g/kg										
Corn	692	692	692	692	692	730	730	730	730	730
Soybean meal	248	248	248	248	248	221	221	221	221	221
Soybean oil	3.00	3.00	3.00	3.00	3.00	1.10	1.10	1.10	1.10	1.10
Common salt	3.50	3.50	3.50	3.50	3.50	3.50	3.50	3.50	3.50	3.50
Limestone	11.8	11.8	11.8	11.8	11.8	11.9	11.9	11.9	11.9	11.9
Dicalcium phosphate	7.90	7.90	7.90	7.90	7.90	8.00	8.00	8.00	8.00	8.00
Vitamin premix^b^	1.00	1.00	1.00	1.00	1.00	1.00	1.00	1.00	1.00	1.00
Mineral premix^c^	0.80	0.80	0.80	0.80	0.80	0.80	0.80	0.80	0.80	0.80
DL-Methionine	0.00	1.60	3.20	4.80	6.40	0.10	1.70	3.30	4.90	6.50
L-Lysine HCl	4.10	4.10	4.10	4.10	4.10	2.70	2.70	2.70	2.70	2.70
L-Glutamate	8.00	8.00	8.00	8.00	8.00	–	–	–	–	–
Threonine	1.30	1.30	1.30	1.30	1.30	0.60	0.60	0.60	0.60	0.60
Arginine	1.10	1.10	1.10	1.10	1.10	0.10	0.10	0.10	0.10	0.10
L-Cystine	–	–	–	–	–	0.10	0.10	0.10	0.10	0.10
Isoleucine	0.20	0.20	0.20	0.20	0.20	–	–	–	–	–
Glycine	10.0	9.20	8.40	7.60	6.80	5.00	4.30	3.50	2.60	1.80
Sand	7.60	6.80	6.00	5.20	4.60	14.5	13.6	12.80	12.10	11.30
Total	1,000	1,000	1,000	1,000	1,000	1,000	1,000	1,000	1,000	1,000
Calculated nutrients (g/kg) and energy										
Crude protein	200	200	200	200	200	170	170	170	170	170
ME, kcal/kg	3,030	3,030	3,030	3,030	3,030	3,030	3,030	3,030	3,030	3,030
Ca	7.00	7.00	7.00	7.00	7.00	7.00	7.00	7.00	7.00	7.00
Available P	2.90	2.90	2.90	2.90	2.90	2.90	2.90	2.90	2.90	2.90
Lysine	11.2	11.2	11.2	11.2	11.2	9.50	9.50	9.50	9.50	9.50
Methionine	2.80	4.40	6.00	7.60	9.20	2.80	4.40	6.00	7.60	9.20
TSAA	5.30	6.90	8.50	10.1	11.7	5.30	6.90	8.50	10.1	11.7
Threonine	7.30	7.30	7.30	7.30	7.30	6.20	6.20	6.20	6.20	6.20
Arginine	11.8	11.8	11.8	11.8	11.8	10.0	10.0	10.0	10.0	10.0
Analyzed amino acids, g/kg										
Crude protein	195	199	211	211	204	177	182	176	181	176
Lysine	13.1	12.5	12.7	13.0	13.2	10.5	11.4	11.4	11.6	11.3
Methionine	2.90	4.50	5.90	7.80	8.90	2.90	4.50	6.30	7.20	8.80
TSAA	5.90	7.40	9.10	10.9	11.5	5.80	7.60	9.20	10.3	11.8
Threonine	7.60	7.60	7.70	7.60	7.70	6.50	7.10	7.00	7.10	7.00
Arginine	12.1	11.7	11.6	12.4	12.5	10.1	11.3	11.0	11.5	10.6
Glutamate	39.2	37.3	37.3	39.3	39.2	30.2	32.7	32.2	33.3	31.5
Amino acids to lysine ratios										
Lysine	100	100	100	100	100	100	100	100	100	100
Methionine	22.2	34.1	46.5	59.8	67.4	27.1	39.5	54.8	62.1	77.9
TSAA	45.2	56.3	71.7	83.8	90.5	54.8	66.7	80.7	88.8	104
Threonine	57.9	60.4	60.6	58.3	58.0	61.4	62.3	60.9	61.2	61.5
Arginine	92.7	89.3	91.3	95.7	94.7	96.2	99.1	96.5	98.7	93.8
Glutamate	300	286	294	303	297	288	287	282	287	278

^a^*NCP* Normal protein diet with 20% crude protein content, *LCP* Reduced protein diet with 17% crude protein content, *Met 2.80* Diet containing 2.80 g/kg of methionine, *Met 4.40* Diet containing 4.40 g/kg of methionine, *Met 6.00* Diet containing 6.00 g/kg of methionine, *Met 7.60* Diet containing 7.60 g/kg of methionine, *Met 9.20* Diet containing 9.20 g/kg of methionine, *TSAA* Total sulfur amino acids

^b^Supplemented per kg of diet: thiamin mononitrate, 2.4 mg; nicotinic acid, 44 mg; riboflavin, 4.4 mg; D-Ca pantothenate, 12 mg; vitamin B_12_ (cobalamin), 12.0 g; pyridoxine HCl, 4.7 mg; D-biotin, 0.11 mg; folic acid, 5.5 mg; menadione sodium bisulfite complex, 3.34 mg; choline chloride, 220 mg; cholecalciferol, 27.5 g; *trans*-retinyl acetate, 1,892 g; *α* tocopheryl acetate, 11 mg; ethoxyquin, 125 mg

^c^Supplemented per kg of diet: manganese (MnSO_4_·H_2_O), 60 mg; iron (FeSO_4_·7H_2_O), 30 mg; zinc (ZnO), 50 mg; copper (CuSO_4_·5H_2_O), 5 mg; iodine (ethylenediamine dihydroiodide), 0.15 mg; selenium (NaSeO_3_), 0.3 mg

### Growth performance and sample collection

Body weight (BW) was measured on 0, 6, and 9 days post inoculation (DPI) for the calculation of body weight gain (BWG). Feed intake (FI) was measured daily after inoculation. Feed conversion ratio (FCR) was calculated from BWG and FI. Mortality was monitored and recorded daily. On 6 and 9 DPI, one bird per cage was randomly selected and euthanized for sample collections. Samples from liver, jejunal mucosa, and cecal tonsils (CT) were collected and snap-frozen in liquid nitrogen. The samples were stored at –80 °C for oxidative status and gene expression analyses. Approximately 2 cm in length of the duodenum, jejunum, and ileum samples were collected, rinsed with PBS, and fixed in 10% formalin for intestinal morphology analysis. Excreta were collected daily from 1 to 9 DPI from each cage for the measurement of oocyst shedding.

### Intestinal morphology and intestinal permeability

The intestinal samples were removed from the 10% formalin after fixation and subsequently embedded in paraffin blocks. The sample blocks were sliced into 4 μm sections and stained with hematoxylin and eosin. The image of the stained tissues was observed and captured under a light microscope with 2 × magnification (BZ-X800, Keyence Inc., Itasca, IL, USA). The villus height (VH) and crypt depth (CD) were measured and VH:CD ratio were calculated as described previously [4]. On 5 DPI, one bird per cage was gavaged with 1 mL of fluorescein isothiocyanate dextran (FITC-d; 2.2 mg/mL, MW 4000; Sigma-Aldrich, St. Louis, MO, USA) to measure intestinal permeability. Two hours after the gavage, the birds were euthanized for blood collection. The blood was centrifuged at 1,000 × *g* for 15 min (Eppendorf Centrifuge 5430R, Eppendorf, Hamburg, Germany), and 100 μL of serum was used to determine the FITC-d concentration according to the method described previously [48].

### Oocyst shedding

The oocyst shedding was measured according to a previously reported method [49]. Briefly, 5 g of the collected excreta samples were weighed and combined with 25 mL of water. The mixture was vigorously vortexed, and 1 mL of the diluted samples was then mixed with 9 mL of saturated salt solution and vortexed thoroughly. The prepared samples were loaded into a McMaster chamber (Jorgensen Laboratories, Loveland, CO, USA) and examined under a microscope (FEC Source, Grand Ronde, OR, USA). The oocysts shedding of *E. acervulina*, *E. maxima*, and *E. tenella* were distinguished by their distinct oocyst sizes and shapes [50]. The oocysts were quantified, and the results were expressed as the log_10_ of oocysts per gram of excreta (OPG).

### Oxidative status analyses

Concentrations of malondialdehyde (MDA), glutathione (GSH), glutathione disulfide (GSSG), and activities of glutathione peroxidase (GPX) and superoxide dismutase (SOD) in the liver were determined using commercial assay kits (GSH, GPX, SOD assay kits, Cayman chemical, Ann Arbor, MI, USA; MDA, BioAssay Systems, Hayward, CA, USA). Protein concentrations of the liver samples were measured by bicinchoninic acid assay (BCA) kit (Thermo Scientific, Rockford, IL, USA) to standardize the results obtained as described previously [51].

### Reverse transcription and real-time PCR analysis

Liver, jejunal mucosa, and CT samples were homogenized with a MiniBeadBeater-16 (BioSpec Products Inc., Bartlesville, OK, USA), and RNA was extracted using QIAzol Lysis Reagents (Qiagen, Germantown, MD, USA) following the manufacturer’s instructions. RNA concentrations were determined by a NanoDrop 2000 spectrophotometer (Thermo Fisher Scientific, MA, USA). The extracted RNA was diluted to a uniform concentration and reverse-transcribed to cDNA by high-capacity cDNA synthesis kits (Applied Biosystems, Forester City, CA, USA). The cDNA samples were combined with SYBR Green Master Mix (Bio-Rad Laboratories, Hercules, CA, USA) and reverse and forward primers for the real-time PCR analysis performed in a Step One thermocycler (Applied Biosystems, Foster City, CA, USA). Primer sequences for tested genes are listed in Table 2. The 2^−ΔΔCt^ method was used to analyze target gene expression over the housekeeping gene, β-actin [52].

**Table 2:** List of primer sequences used for real-time PCR

Gene symbol^a^	Accession number	Forward primer (5´→3´)	Reverse primer (5´→3´)
Beta-actin^b^	NM_205518.2	CAACACAGTGCTGTCTGGTGGTA	ATCGTACTCCTGCTTGCTGATCC
*MUC2*	XM_040673077.2	ATGCGATGTTAACACAGGACTC	GTGGAGCACAGCAGACTTTG
*OCLN*	NM_205128.1	ACGGCAGCACCTACCTCAA	GGCGAAGAAGCAGATGAG
*CLDN1*	NM_001013611.2	TGGAGGATGACCAGGTGAAGA	CGAGCCACTCTGTTGCCATA
*ZO2*	NM_001396728.1	GGCAAATCATTGAGCAGGA	ATTGATGGTGGCTGTAAAGAG
*SLC6A19*	XM_419056.5	TCTATTGAAGATTCGGGCAC	AATGGTAAGCACAAGGTATGG
*SLC7A9*	XM_046925532.1	GCATCTTTGTTTCCCCAAAA	AGCTTGCCCAAGAAAACAGA
*SLC43A2*	XM_415803.6	CCTGTCTCATTCCCAACCTAC	CTGCAACCCTGTCAAGCTAC
*IL1β*	NM_204524.2	TGCCTGCAGAAGAAGCCTCG	GACGGGCTCAAAAACCTCCT
*TNFα*	MF000729.1	CGTGGTTCGAGTCGCTGTAT	CCGTGCAGGTCGAGGTACT
*IFNγ*	NM_205149.2	CACATATCTGAGGAGCTCTATAC	GTTCATTCGCGGCTTTG
*IL10*	NM_001004414.4	AGCAGATCAAGGAGACGTTC	ATCAGCAGGTACTCCTCGAT
*TGFβ1*	NM_001318456.1	ATGAGTATTGGGCCAAAG	ACGTTGAACACGAAGAAG
*MAT1A*	NM_001199519.2	TCATACCAGTGCGTGTCCAT	CTGAGGCCCTCCAATAACAA
*AHCYL1*	XM_040652696.2	GGAAGCAAGTGGTGGTTTGT	CTTCATCCGATCCAGGTGTT
*MTR*	NM_001396228.1	TACACCGGCACATATCAGGA	TGGCTACAGTCAGGGCTTCT
*CBS*	XM_040659743.2	TACCATCACTGGCATCTCCA	TGCGTGCTAAAGCAAATGAC
*MTHFR*	NM_001328491.2	ACTGAAGTCCTTAAGCGCCT	GGAGTTACCCCATCGACCAT
*DHFR*	NM_001006584.3	CCAGAGAATGACCAGCACCT	GCCTCCAACAATCCAAACCA
*SHMT*	XM_040683445.2	ACTGGGATCCTGCTTGAACA	ACGTTGACACCCCACTTTTG
*MTHFD1*	NM_001039303.2	GTTTGGTGCCTTCGGTCAAT	ATGTTATGGTGGCTGGGTCA
*TYMS*	XM_046912181.1	CGCGGTACAGTCTGAGAGAT	AGGAACTCACGTGACCCATT

^a^*MUC2* Mucin 2, *OCLN* Occludin, *CLDN1* Claudin 1, *ZO2* Zonula occludens 2, *SLC6A19* Sodium-dependent neutral amino acid transporter B(0)AT1, *SLC7A9* b(0, +)-type amino acid transporter 1, *SLC43A2* L-type amino acid transporter 4, *IL1β* Interleukin 1 beta, *TNFα* Tumor necrotic factor alpha, *IFNγ* Interferon gamma, *IL10* Interleukin 10, *TGFβ* Transforming growth factor beta, *MAT1A* Methionine adenosyltransferase 1A, *AHCYL1* Adenosylhomocysteinase like 1, *MTR* Methionine synthase, *CBS* Cystathionine beta synthase, *MTHFR* Methylenetetrahydrofolate reductase, *DHFR* Dihydrofolate reductase, *SHMT* Serine hydroxymethyltransferase, *MTHFD1* Methylenetetrahydrofolate dehydrogenase 1, *TYMS* Thymidylate synthase

^b^Housekeeping gene

### Statistical analysis

Statistical analysis was conducted by the PROC GLM program of SAS software (SAS Institute Inc., Cary, NC, USA). The FI of each DPI were analyzed separately by two-way ANOVA in the 2 × 5 factorial arrangement with CP and Met levels being the two main effects. The accumulated FI of each period and the growth performance parameters, intestinal morphology, oxidative status, and gene expression data were analyzed by two-way ANOVA in the same 2 × 5 factorial arrangement. Tukey’s honestly significant difference test was applied to separate means. Linear and quadratic orthogonal polynomial contrasts were utilized to evaluate the effects of Met levels of the tested parameters. Statistical significance was set at *P* ≤ 0.05.

## Result

### Growth performance

**Fig. 1:**
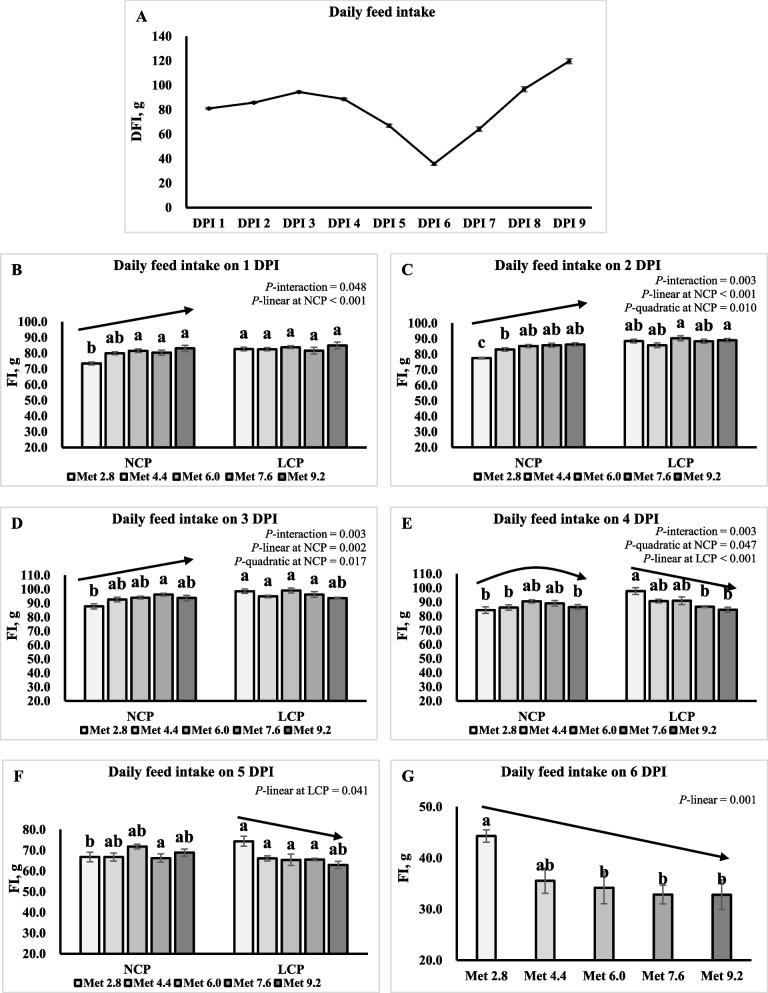
Effects of dietary methionine levels and protein contents on the daily feed intake of broilers challenged with *Eimeria* spp. The error bars represent the SEM values. Bars without a common letter differ significantly. The black lines with arrowhead represented significant linear or quadratic relationship between parameters and dietary methionine levels. Statistical significance was set at *P* ≤ 0.05. DFI, Daily feed intake; DPI, Day post inoculation; Met, Methionine; NCP, Normal protein diet; LCP, Reduced protein diet; Met 2.8, Dietary Met level = 2.8 g/kg; Met 4.4, Dietary Met level = 4.4 g/kg; Met 6.0, Dietary Met level = 6.0 g/kg; Met 7.6, Dietary Met level = 7.6 g/kg; Met 9.0, Dietary Met level = 9.0 g/kg

The daily FI began to decline from 4 DPI, reaching its lowest point on 6 DPI. Subsequently, it gradually increased from 6 to 9 DPI (Fig. 1A). Significant interaction effects were observed for the daily FI of 1–3 DPI (*P* < 0.05) (Table 3). Specifically, the daily FI linearly or quadratically increased as Met levels increased in the NCP groups (*P* < 0.01), whereas they were not affected by Met levels in the LCP groups (Fig. 1B–D). A significant interaction effect was observed for the daily FI of 4 DPI (*P* < 0.01). Specifically, as Met level increased, daily FI changed quadratically in the NCP groups (*P* < 0.05), whereas it linearly decreased in the LCP groups (*P* < 0.01) (Fig. 1E). On 5 DPI, daily FI linearly decreased as Met levels increased in the LCP groups (*P* < 0.05) (Fig. 1F). The daily FI of 6 DPI linearly decreased as Met levels increased (*P* < 0.01) (Fig. 1G) and it was significantly higher in the Met 2.8 groups than the Met 6.0, Met 7.6, and Met 9.2 groups (*P* < 0.01).

**Table 3:** Effects of dietary methionine levels and protein contents on post inoculation daily feed intake of broilers under *Eimeria* challenge

Items^1^		1 DPI	2 DPI	3 DPI	4 DPI	5 DPI	6 DPI	7 DPI	8 DPI	9 DPI
Main effect of protein content^2^										
NCP		79.6^b^	83.6^b^	92.8^b^	87.3^b^	68.1	37.9	63.6	99.3	117
LCP		83.1^a^	88.0^a^	96.3^a^	90.2^a^	66.8	33.4	65.8	95.0	119
Main effect of methionine content										
Met 2.8		78.1^b^	83.0^b^	93.1	91.1^a^	70.5	44.3^a^	71.3	103	117
Met 4.4		81.2^ab^	84.4^ab^	93.7	88.5^ab^	66.4	35.5^ab^	62.0	94.8	118
Met 6.0		82.7^a^	87.8^a^	96.5	90.8^ab^	68.6	34.2^b^	62.6	95.8	116
Met 7.6		81.0^ab^	87.1^a^	96.3	87.9^ab^	65.9	32.8^b^	65.5	92.4	114
Met 9.2		84.0^a^	87.6^a^	93.3	85.6^b^	63.9	32.8^b^	61.9	99.4	126
Interaction effect										
NCP	Met 2.8	73.4^b^	77.5^c^	87.7^b^	84.3^b^	66.7	44.3	67.5	99.5	111
NCP	Met 4.4	79.9^a^	83.1^b^	92.6^ab^	86.1^b^	66.7	38.7	64.3	103	120
NCP	Met 6.0	81.5^a^	85.3^ab^	94.0^ab^	90.5^ab^	71.8	38.8	62.4	100	114
NCP	Met 7.6	80.3^a^	85.8^ab^	96.2^a^	89.1^ab^	66.2	31.6	61.3	86.7	110
NCP	Met 9.2	83.0^a^	86.3^ab^	93.6^ab^	86.5^b^	68.9	36.1	62.5	107	131
LCP	Met 2.8	82.7^a^	88.5^ab^	98.5^a^	97.8^a^	74.3	44.3	75.2	107	123
LCP	Met 4.4	82.5^a^	85.8^ab^	94.8^a^	90.8^ab^	66.1	32.3	59.6	86.5	116
LCP	Met 6.0	83.8^a^	90.3^a^	99.0^a^	91.0^ab^	65.4	29.6	62.9	91.6	118
LCP	Met 7.6	81.6^a^	88.4^ab^	96.2^a^	86.7^b^	65.6	34.1	69.8	98.2	117
LCP	Met 9.2	84.9^a^	89.0^a^	93.6^ab^	84.6^b^	62.9	29.6	61.3	91.6	122
*P*-value										
CP		0.003	< 0.001	< 0.001	0.022	0.583	0.067	0.586	0.299	0.641
Met		< 0.001	< 0.001	0.063	0.037	0.607	0.006	0.506	0.483	0.354
CP × Met		0.048	0.003	0.003	< 0.001	0.287	0.361	0.796	0.105	0.475
SEM		1.42	1.19	1.50	1.90	3.76	3.23	6.17	6.31	6.52

^a,b^Means within a column lacking a common superscript differ (*P* < 0.05)

^1^DPI, Days post inoculation

^2^NCP, Normal protein group; LCP, Reduced protein group; Met, Methionine; Met 2.8, Dietary Met level = 2.8 g/kg; Met 4.4, Dietary Met level = 4.4 g/kg; Met 6.0, Dietary Met level = 6.0 g/kg; Met 7.6, Dietary Met level = 7.6 g/kg; Met 9.0, Dietary Met level = 9.0 g/kg

**Fig. 2:**
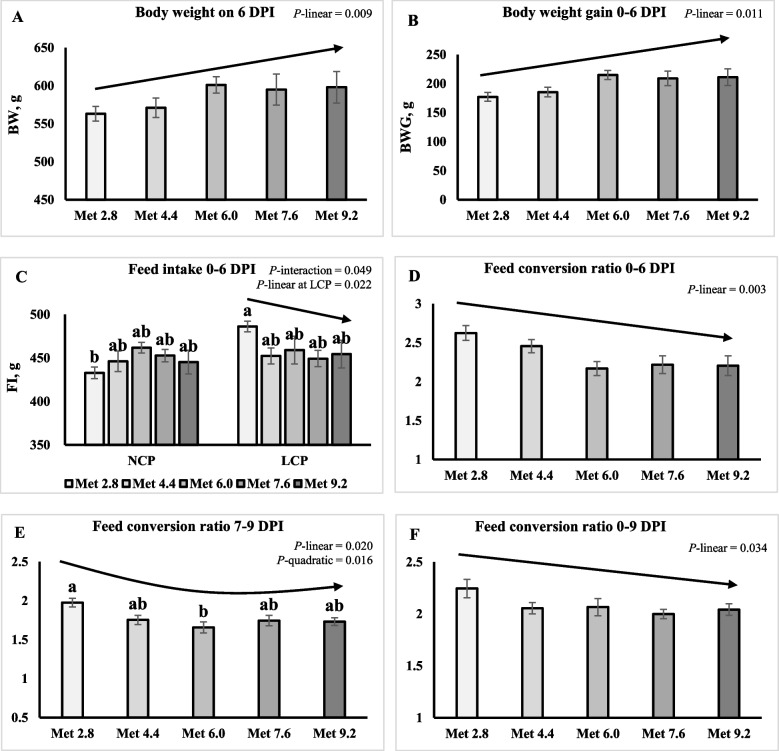
Effects of dietary methionine levels and protein contents on the growth performance of broilers challenged with *Eimeria* spp. The error bars represent the SEM values. Bars without a common letter differ significantly. The black lines with arrowhead represented significant linear or quadratic relationship between parameters and dietary methionine levels. Statistical significance was set at *P* ≤ 0.05. DPI, Day post inoculation; Met, Methionine; NCP, Normal protein diet; LCP, Reduced protein diet; Met 2.8, Dietary Met level = 2.8 g/kg; Met 4.4, Dietary Met level = 4.4 g/kg; Met 6.0, Dietary Met level = 6.0 g/kg; Met 7.6, Dietary Met level = 7.6 g/kg; Met 9.0, Dietary Met level = 9.0 g/kg

On 6 DPI, BW and BWG linearly increased, and FCR linearly decreased as Met level increased (*P* < 0.05) (Fig. 2A–C). An interaction effect was observed for the FI of 0–6 DPI (*P* < 0.05) where it linearly decreased as Met level increased in the LCP groups while not in the NCP groups (Fig. 2D). The FCR of 0–6 DPI was significantly lower in the Met 6.0 groups compared to the Met 2.8 groups (*P* = 0.019) (Table 4). The FCR of 7–9 DPI quadratically decreased as Met level increased (*P* < 0.05) (Fig. 2E), and it was significantly lower in the Met 6.0 groups compared to the Met 2.8 groups (*P* = 0.018). The FCR of 0–9 DPI linearly decreased as Met level increased (*P* < 0.05) (Fig. 2F). No treatment effects were observed for the mortality on any timepoints.

**Table 4:** Effects of dietary methionine levels and protein contents on growth performance of broilers under *Eimeria* challenge

Items^1^		0–6 DPI				7–9 DPI			0–9 DPI			
		BW	BWG	FI	FCR	BWG	FI	FCR	BW	BWG	FI	FCR
Main effect of protein content^2^												
NCP		585	199	449	2.32	161	279	1.78	742	355	724	2.07
LCP		586	200	459	2.35	159	280	1.77	743	357	737	2.09
Main effect of methionine content												
Met 2.8		563	177	459	2.62^a^	152	292	1.98^a^	726	341	752	2.24
Met 4.4		571	185	449	2.46^ab^	158	274	1.75^ab^	742	356	724	2.05
Met 6.0		601	215	460	2.17^b^	164	273	1.66^b^	745	359	736	2.07
Met 7.6		595	209	451	2.22^ab^	159	272	1.75^ab^	748	361	720	2.01
Met 9.2		598	211	450	2.20^ab^	166	288	1.73^ab^	752	365	737	2.04
Interaction effect												
NCP	Met 2.8	553	166	433^b^	2.61	146	278	1.99	702	315	711	2.31
NCP	Met 4.4	569	183	446^ab^	2.47	163	287	1.79	739	353	733	2.10
NCP	Met 6.0	608	222	462^ab^	2.09	172	272	1.61	772	386	733	1.91
NCP	Met 7.6	604	216	449^ab^	2.17	148	258	1.78	730	342	703	2.07
NCP	Met 9.2	593	206	454^ab^	2.26	174	301	1.72	768	381	755	1.98
LCP	Met 2.8	573	188	486^a^	2.63	158	306	1.96	751	366	792	2.18
LCP	Met 4.4	574	188	452^ab^	2.44	152	262	1.71	745	359	714	2.01
LCP	Met 6.0	595	208	459^ab^	2.24	156	274	1.70	718	332	739	2.22
LCP	Met 7.6	587	202	453^ab^	2.26	171	285	1.71	766	381	738	1.94
LCP	Met 9.2	604	216	445^ab^	2.15	159	275	1.75	736	348	720	2.10
*P*-value												
CP		0.901	0.862	0.146	0.781	0.858	0.929	0.835	0.932	0.902	0.381	0.744
Met		0.051	0.059	0.723	0.019	0.865	0.706	0.018	0.836	0.859	0.693	0.110
CP × Met		0.712	0.730	0.049	0.931	0.528	0.386	0.862	0.153	0.128	0.122	0.095
SEM		15.4	15.3	10.2	0.154	14.1	18.1	0.089	23.0	22.9	22.6	0.092

^a,b^Means within a column lacking a common superscript differ (*P* < 0.05)

^1^DPI, Days post inoculation; BW, Body weight (g); BWG, Body weight gain (g); FI, Feed intake (g)

^2^NCP, Normal protein group; LCP, Reduced protein group; Met, Methionine; Met 2.8, Dietary Met level = 2.8 g/kg; Met 4.4, Dietary Met level = 4.4 g/kg; Met 6.0, Dietary Met level = 6.0 g/kg; Met 7.6, Dietary Met level = 7.6 g/kg; Met 9.0, Dietary Met level = 9.0 g/kg

### Intestinal morphology

**Fig. 3:**
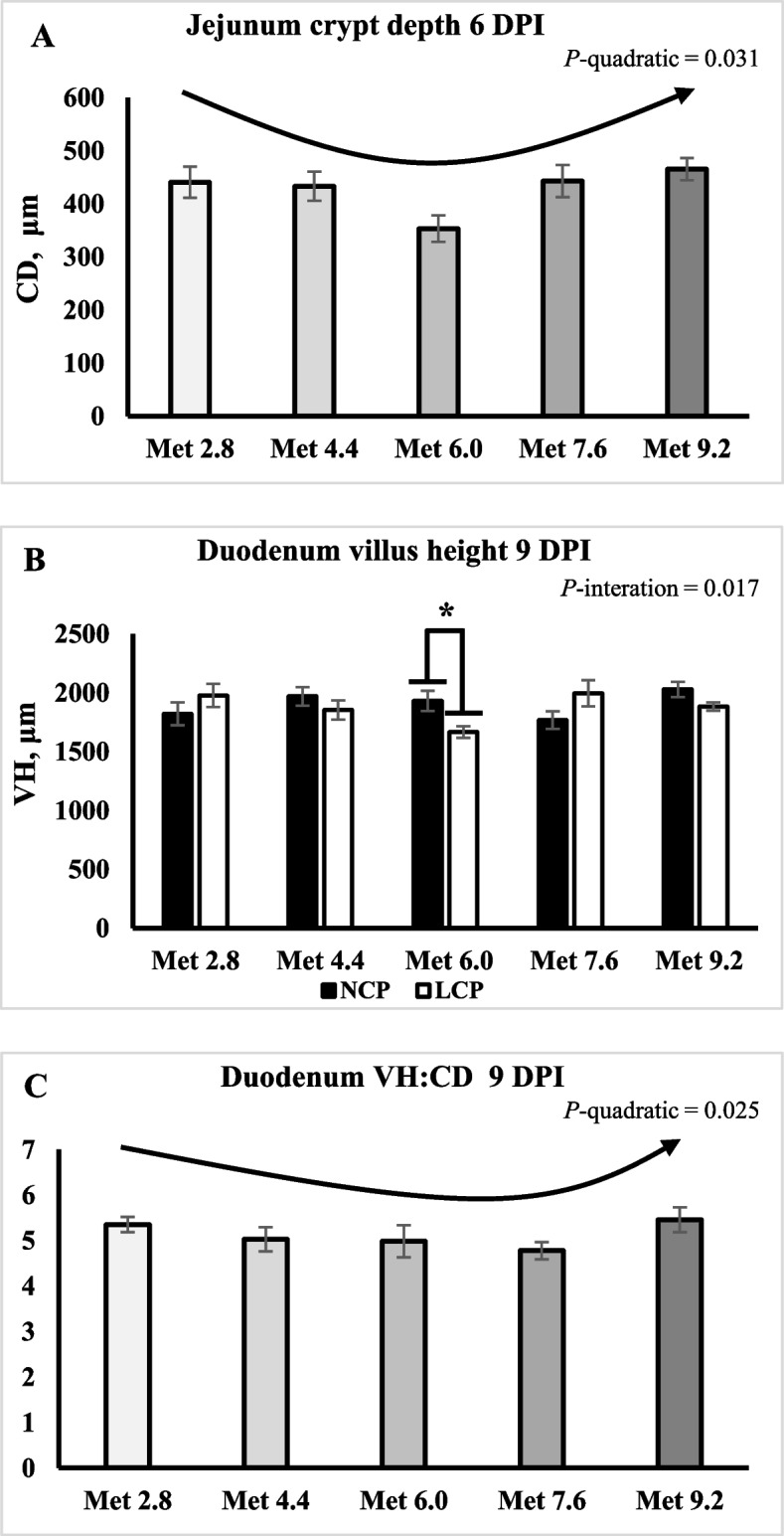
Effects of dietary methionine levels and protein contents on intestinal morphology of broilers challenged with *Eimeria* spp. The error bars represent the SEM values. Bars without a common letter differ significantly. The asterisk (*) denotes significant differences. The black lines with arrowhead represented significant linear or quadratic relationship between parameters and dietary methionine levels. Statistical significance was set at *P* ≤ 0.05. DPI, Day post inoculation; CD, Crypt depth; VH, Villus height; Met, Methionine; NCP, Normal protein diet; LCP, Reduced protein diet; Met 2.8, Dietary Met level = 2.8 g/kg; Met 4.4, Dietary Met level = 4.4 g/kg; Met 6.0, Dietary Met level = 6.0 g/kg; Met 7.6, Dietary Met level = 7.6 g/kg; Met 9.0, Dietary Met level = 9.0 g/kg

On 6 DPI, the duodenal CD was lower in the LCP groups compared to the NCP groups (*P* < 0.01) (Table 5). The duodenal VH:CD ratio was higher in the LCP groups than in the NCP groups (*P* < 0.01). On 6 DPI, the jejunal CD changed quadratically as it decreased initially and then increased as Met level increased (*P* < 0.05) (Fig. 3A). On 9 DPI, the duodenal VH:CD ratio was lower in the LCP groups than in the NCP groups (*P* < 0.05) (Table 6). An interaction effect was observed for the duodenal VH (*P* < 0.05), where the LCP diet significantly decreased VH only in the Met 6.0 group. (Fig. 3B). The VH:CD ratio changed quadratically as it decreased initially and then increased as Met level increased (*P* < 0.05) (Fig. 3C). No significant effects on the ileal morphology were observed on 6 and 9 DPI.

**Table 5:** Effects of dietary methionine levels and protein contents on intestinal morphology of broilers challenged with *Eimeria* spp. on 6 day post inoculation

Items^1^		Duodenum			Jejunum			Ileum		
		VH	CD	VH:CD	VH	CD	VH:CD	VH	CD	VH:CD
Main effect of protein content^2^										
NCP		1,522	470^b^	2.91^b^	711	440	1.68	536	327	1.59
LCP		1,652	528^a^	3.61^a^	750	414	1.83	598	309	1.80
Main effect of methionine content										
Met 2.8		1,562	491	3.28	694	441	1.59	538	309	1.61
Met 4.4		1,561	478	3.41	715	433	1.70	606	328	1.74
Met 6.0		1,683	486	3.48	768	353	1.97	549	296	1.65
Met 7.6		1,655	533	3.20	756	443	1.84	566	352	1.72
Met 9.2		1,473	508	2.91	717	465	1.68	575	306	1.75
Interaction effect										
NCP	Met 2.8	1,531	521	2.97	668	422	1.60	505	291	1.70
NCP	Met 4.4	1,420	522	2.84	682	453	1.56	557	359	1.69
NCP	Met 6.0	1,604	505	3.14	720	381	1.74	516	304	1.44
NCP	Met 7.6	1,582	580	2.77	755	460	1.77	550	381	1.51
NCP	Met 9.2	1,472	513	2.82	728	485	1.74	552	302	1.62
LCP	Met 2.8	1,593	462	3.60	719	459	1.58	570	327	1.51
LCP	Met 4.4	1,702	433	3.98	748	413	1.84	656	297	1.80
LCP	Met 6.0	1,762	466	3.82	817	326	2.21	582	289	1.86
LCP	Met 7.6	1,728	486	3.63	757	426	1.90	582	324	1.93
LCP	Met 9.2	1,475	503	3.00	707	446	1.62	598	310	1.88
*P*-value										
CP		0.101	0.003	0.007	0.306	0.294	0.361	0.072	0.337	0.126
Met		0.477	0.332	0.641	0.703	0.062	0.622	0.735	0.336	0.947
CP × Met		0.823	0.565	0.792	0.861	0.786	0.793	0.976	0.375	0.570
SEM		122	28.7	0.386	59.4	38.9	0.254	52.4	29.0	0.256

^a,b^Means within a column lacking a common superscript differ (*P* < 0.05)

^1^VH, Villus height (μm); CD, Crypt depth (μm)

^2^NCP, Normal protein group; LCP, Reduced protein group; Met, Methionine; Met 2.8, Dietary Met level = 2.8 g/kg; Met 4.4, Dietary Met level = 4.4 g/kg; Met 6.0, Dietary Met level = 6.0 g/kg; Met 7.6, Dietary Met level = 7.6 g/kg; Met 9.0, Dietary Met level = 9.0 g/kg

**Table 6:** Effects of dietary methionine levels and protein contents on intestinal morphology of broilers challenged with *Eimeria* spp. on 9 day post inoculation

Items^1^		Duodenum			Jejunum			Ileum		
		VH	CD	VH:CD	VH	CD	VH:CD	VH	CD	VH:CD
Main effect of protein content^2^										
NCP		1903	360	5.36^a^	879	317	2.73	774	268	2.94
LCP		1874	367	4.83^b^	897	322	2.80	772	252	3.12
Main effect of methionine content										
Met 2.8		1899	356	5.35	915	357	2.71	786	272	2.79
Met 4.4		1910	375	5.00	907	308	2.84	768	250	3.19
Met 6.0		1798	361	4.89	829	334	2.63	770	265	2.91
Met 7.6		1881	361	4.78	901	302	2.90	732	278	2.79
Met 9.2		1955	363	5.46	888	296	2.74	807	235	3.47
Interaction effect										
NCP	Met 2.8	1821	334	5.46	898	366	2.68	746	265	2.88
NCP	Met 4.4	1968	379	5.25	905	323	2.51	744	275	2.79
NCP	Met 6.0	1931	339	5.70	826	322	2.74	817	261	2.92
NCP	Met 7.6	1768	375	4.76	902	293	2.85	744	305	2.61
NCP	Met 9.2	2028	370	5.62	865	282	2.86	818	234	3.52
LCP	Met 2.8	1977	379	5.24	933	349	2.73	826	278	2.70
LCP	Met 4.4	1853	370	4.75	910	293	3.18	791	224	3.60
LCP	Met 6.0	1666	382	4.08	831	347	2.53	724	269	2.89
LCP	Met 7.6	1995	347	4.80	900	311	2.94	720	251	2.97
LCP	Met 9.2	1882	357	5.29	910	310	2.62	797	237	3.43
*P*-value										
CP		0.575	0.630	0.017	0.612	0.840	0.701	0.944	0.287	0.421
Met		0.389	0.956	0.200	0.476	0.398	0.891	0.547	0.348	0.198
CP × Met		0.017	0.420	0.152	0.988	0.896	0.567	0.347	0.405	0.561
SEM		79.7	24.1	0.333	53.2	35.6	0.292	44.7	23.2	0.336

^a,b^ Means within a column lacking a common superscript differ (*P* < 0.05)

^1^VH, Villus height (μm); CD, crypt depth (μm)

^2^NCP, Normal protein group; LCP, Reduced protein group; Met, Methionine; Met 2.8, Dietary Met level = 2.8 g/kg; Met 4.4, Dietary Met level = 4.4 g/kg; Met 6.0, Dietary Met level = 6.0 g/kg; Met 7.6, Dietary Met level = 7.6 g/kg; Met 9.0, Dietary Met level = 9.0 g/kg

### Gene expression of tight junction proteins

**Fig. 4:**
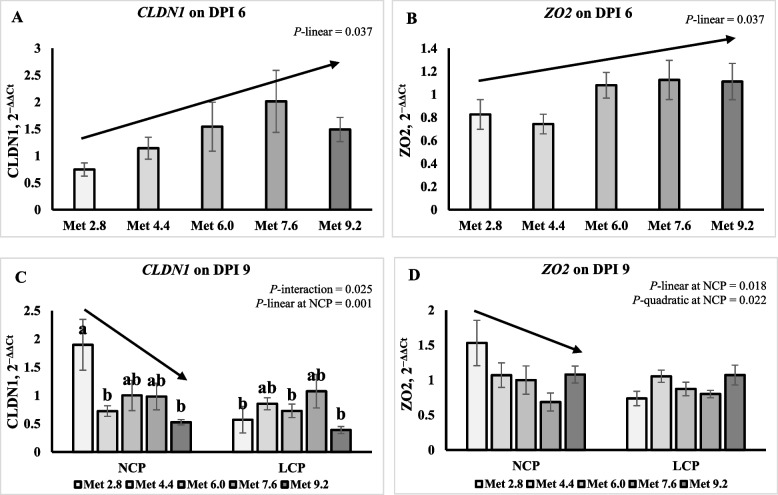
Effects of dietary methionine levels and protein contents on expression of tight junction proteins of broilers challenged with *Eimeria* spp. The error bars represent the SEM values. Bars without a common letter differ significantly. The black lines with arrowhead represented significant linear or quadratic relationship between parameters and dietary methionine levels. Statistical significance was set at *P* ≤ 0.05. DPI, Day post inoculation; *CLDN1*, Claudin-1; *ZO2*, Zonula occludens 2; Met, Methionine; NCP, Normal protein diet; LCP, Reduced protein diet; Met 2.8, Dietary Met level = 2.8 g/kg; Met 4.4, Dietary Met level = 4.4 g/kg; Met 6.0, Dietary Met level = 6.0 g/kg; Met 7.6, Dietary Met level = 7.6 g/kg; Met 9.0, Dietary Met level = 9.0 g/kg

On 6 DPI, the expression of claudin-1 (*CLDN1*) was higher in the LCP groups than in the NCP groups (*P* < 0.01) (Table 7). The expression of *CLDN1* and zonula occludens (*ZO2*) linearly increased as Met level increased (*P* < 0.05) (Fig. 4A and B). On 9 DPI, an interaction effect was observed for the expression of* CLDN1*( *P* < 0.05), where it linearly decreased as Met level increased in the NCP groups while not in the LCP groups (Fig. 4C). The expression of *ZO2* quadratically decreased as Met level increased (*P* < 0.05) in the NCP groups (Fig. 4D). No significant CP or Met main effects were observed for the expression of tight junction protein on 9 DPI (Table 8).

**Table 7:** Effects of dietary methionine levels and protein contents on expression of tight junction proteins and amino acid transporters of broilers challenged with *Eimeria* spp. on 6 day post inoculation

Items^1^		*MUC2*	*OCLN*	*ZO2*	*CLDN1*	*SLC6A19*	*SLC7A9*	*SLC43A2*
Main effect of protein content^2^								
NCP		1.15	0.95	0.97	1.01^a^	1.10	1.16^a^	1.07^a^
LCP		0.98	0.97	0.99	1.94^b^	0.87	0.76^b^	0.75^b^
Main effect of methionine content								
Met 2.8		1.18	0.76	0.83	0.75^b^	1.33	1.28	1.14
Met 4.4		1.22	0.77	0.74	1.14^ab^	1.19	1.19	1.00
Met 6.0		0.79	1.15	1.09	1.95^ab^	0.76	0.78	0.79
Met 7.6		1.11	1.16	1.13	2.01^a^	0.79	0.70	0.87
Met 9.2		1.01	0.96	1.11	1.51^ab^	0.85	0.83	0.78
Interaction effect								
NCP	Met 2.8	1.26	0.62	0.77	0.72	1.58	1.83	1.32
NCP	Met 4.4	1.06	0.90	0.86	0.80	0.87	1.02	0.88
NCP	Met 6.0	1.00	1.00	1.00	1.00	1.00	1.00	1.00
NCP	Met 7.6	1.18	1.36	1.13	1.20	0.98	0.82	1.16
NCP	Met 9.2	1.25	0.87	1.08	1.32	1.06	1.14	1.02
LCP	Met 2.8	1.11	0.90	0.89	1.57	1.08	0.73	0.95
LCP	Met 4.4	1.38	0.63	0.63	0.71	1.51	1.36	1.11
LCP	Met 6.0	0.59	1.31	1.18	2.90	0.53	0.57	0.58
LCP	Met 7.6	1.04	0.96	1.12	2.83	0.61	0.57	0.59
LCP	Met 9.2	0.77	1.05	1.15	1.71	0.63	0.51	0.53
*P*-value								
CP		0.263	0.873	0.852	0.002	0.153	0.018	0.025
Met		0.419	0.086	0.181	0.026	0.081	0.132	0.443
CP × Met		0.503	0.208	0.864	0.145	0.116	0.126	0.396
SEM		0.242	0.187	0.200	0.425	0.244	0.263	0.219

^a,b^Means within a column lacking a common superscript differ (*P* < 0.05)

^1^*MUC2* Mucin 2, *OCLN* Occludin, *CLDN1* Claudin 1, *ZO2* Zonula occludens 2, *SLC6A19* Sodium-dependent neutral amino acid transporter B(0)AT1, *SLC7A9* b(0, +)-type amino acid transporter 1, *SLC43A2* L-type amino acid transporter 4

^2^NCP, Normal protein group; LCP, Reduced protein group; Met, Methionine; Met 2.8, Dietary Met level = 2.8 g/kg; Met 4.4, Dietary Met level = 4.4 g/kg; Met 6.0, Dietary Met level = 6.0 g/kg; Met 7.6, Dietary Met level = 7.6 g/kg; Met 9.0, Dietary Met level = 9.0 g/kg

**Table 8:** Effects of dietary methionine levels and protein contents on expression of tight junction proteins and amino acid transporters of broilers challenged with *Eimeria* spp. on 9 day post inoculation

Items^1^		*MUC2*	*OCLN*	*ZO2*	*CLDN1*	*SLC6A19*	*SLC7A9*	*SLC43A2*
Main effect of protein content^2^								
NCP		1.13	1.10	1.07	1.03^a^	1.44	1.00	1.03
LCP		1.15	0.92	0.91	0.72^b^	1.91	1.11	1.12
Main effect of methionine content								
Met 2.8		1.12	1.20	1.13	1.23^a^	2.25	1.21	1.17
Met 4.4		1.21	1.09	1.06	0.79^ab^	1.97	0.98	1.11
Met 6.0		1.11	0.87	0.94	0.86^ab^	1.50	1.05	1.01
Met 7.6		1.21	0.85	0.74	1.03^ab^	1.79	1.04	1.09
Met 9.2		1.05	1.03	1.08	0.46^b^	0.87	0.98	1.01
Interaction effect								
NCP	Met 2.8	1.16	1.47	1.53	1.90^a^	2.08	1.39	0.99
NCP	Met 4.4	1.19	1.09	1.07	0.72^b^	1.83	0.86	0.93
NCP	Met 6.0	1.00	1.00	1.00	1.00^ab^	1.00	1.00	1.00
NCP	Met 7.6	1.17	0.85	0.69	0.98^ab^	1.35	0.80	1.06
NCP	Met 9.2	1.13	1.07	1.08	0.53^b^	0.94	0.94	1.18
LCP	Met 2.8	1.08	0.92	0.74	0.57^b^	2.43	1.03	1.36
LCP	Met 4.4	1.23	1.09	1.06	0.85^ab^	2.11	1.10	1.30
LCP	Met 6.0	1.21	0.74	0.87	0.73^b^	2.00	1.11	1.02
LCP	Met 7.6	1.25	0.85	0.80	1.08^ab^	2.23	1.27	1.11
LCP	Met 9.2	0.98	0.98	1.07	0.39^b^	0.81	1.03	0.83
*P*-value								
CP		0.863	0.184	0.136	0.047	0.120	0.331	0.359
Met		0.882	0.434	0.192	0.034	0.060	0.718	0.792
CP × Met		0.875	0.663	0.094	0.025	0.764	0.255	0.131
SEM		0.181	0.227	0.164	0.226	0.470	0.182	0.151

^a,b^Means within a column lacking a common superscript differ (*P* < 0.05)

^1^*MUC2* Mucin 2, *OCLN* Occludin, *CLDN1* Claudin 1, *ZO2* Zonula occludens 2, *SLC6A19* Sodium-dependent neutral amino acid transporter B(0)AT1, *SLC7A9* b(0, +)-type amino acid transporter 1, *SLC43A2* L-type amino acid transporter 4

^2^NCP, Normal protein group; LCP, Reduced protein group; Met, Methionine; Met 2.8, Dietary Met level = 2.8 g/kg; Met 4.4, Dietary Met level = 4.4 g/kg; Met 6.0, Dietary Met level = 6.0 g/kg; Met 7.6, Dietary Met level = 7.6 g/kg; Met 9.0, Dietary Met level = 9.0 g/kg

### Gene expression of amino acid transporters

**Fig. 5:**
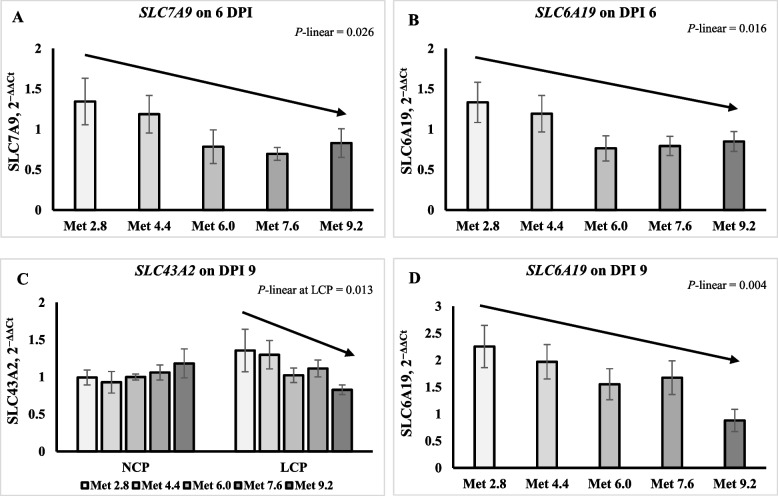
Effects of dietary methionine levels and protein contents on expression of amino acid transporters of broilers challenged with *Eimeria* spp. The error bars represent the SEM values. Bars without a common letter differ significantly. The black lines with arrowhead represented significant linear or quadratic relationship between parameters and dietary methionine levels. Statistical significance was set at *P* ≤ 0.05. DPI, day post inoculation; *SLC7A9*, b(0, +)-type amino acid transporter 1; *SLC6A19*, Sodium-dependent neutral amino acid transporter B(0)AT1; *SLC43A2*, L-type amino acid transporter 4; Met, Methionine; NCP, Normal protein diet; LCP, Reduced protein diet; Met 2.8, Dietary Met level = 2.8 g/kg; Met 4.4, Dietary Met level = 4.4 g/kg; Met 6.0, Dietary Met level = 6.0 g/kg; Met 7.6, Dietary Met level = 7.6 g/kg; Met 9.0, Dietary Met level = 9.0 g/kg

On 6 DPI, the expression of b(0, +)-type amino acid transporter 1 (*SLC7A9*) and L-type amino acid transporter 4 (*SLC43A2*) was lower in the LCP groups than in the NCP groups (*P* < 0.05) (Table 7). The expression of sodium-dependent neutral amino acid transporter B(0)AT1 (*SLC6A19*) and *SLC7A9* linearly decreased as Met level increased (*P* < 0.05) (Fig. 5A and B). On 9 DPI, the expression of *SLC43A2* in the LCP groups and *SLC6A19* linearly decreased as Met level increased (*P* < 0.05) (Fig. 5C and D). No significant effects were observed for the expression of amino acid transporters on 9 DPI (Table 8).

### Gene expression of cytokines

**Fig. 6:**
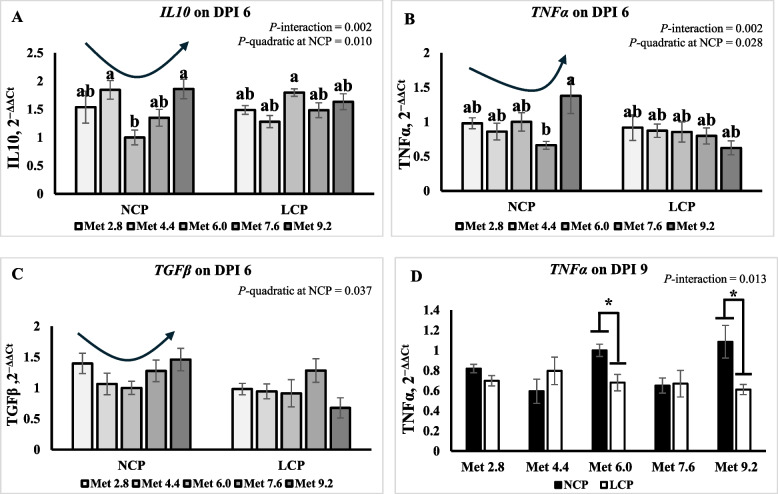
Effects of dietary methionine levels and protein contents on expression of cytokines of broilers challenged with *Eimeria* spp. The error bars represent the SEM values. Bars without a common letter differ significantly. The asterisk (*) denotes significant differences. The black lines with arrowhead represented significant linear or quadratic relationship between parameters and dietary methionine levels. Statistical significance was set at *P* ≤ 0.05. DPI, Day post inoculation; *IL1β*, Interleukin-1β; *IL10*, Interleukin-10; *TNFα*, Tumor necrosis factor α; *TGFβ*, Transforming growth factor β; Met, Methionine; NCP, Normal protein diet; LCP, Reduced protein diet; Met 2.8, Dietary Met level = 2.8 g/kg; Met 4.4, Dietary Met level = 4.4 g/kg; Met 6.0, Dietary Met level = 6.0 g/kg; Met 7.6, Dietary Met level = 7.6 g/kg; Met 9.0, Dietary Met level = 9.0 g/kg

On 6 DPI, the expression of interleukin-1β (*IL1β*) was higher in the Met 4.4 group than in the Met 2.8 and Met 6.0 groups (*P* < 0.01) (Table 9). The expression of transforming growth factor β (*TGFβ*) was higher in the NCP groups than in the LCP groups (*P* < 0.05). Significant interaction effects were observed for the expression of interleukin-10 (*IL10*) and tumor necrosis factor α (*TNFα*) where their expression exhibited a quadratic trend, decreasing initially and then increasing as Met level increased in the NCP groups while not in the LCP groups (Fig. 6A and B). The expression of *TGFβ* exhibited a quadratic trend (*P* < 0.05), decreasing initially and then increasing as Met level increased in the NCP groups (Fig. 6C). On 9 DPI, the expression of *IL10* was higher in the NCP groups than the LCP groups (*P* < 0.05) (Table 9). An interaction effect was observed for the expression of *TNFα* (*P* < 0.05), where LCP diet decreased its expression only in the Met 6.0 and Met 9.2 groups (Fig. 6D).

**Table 9:** Effects of dietary methionine levels and protein contents on expression of cytokines of broilers challenged with *Eimeria* spp.

Items^1^		6 DPI					9 DPI				
		*IL1β*	*IL10*	*INFy*	*TGFβ*	*TNFα*	*IL1β*	*IL10*	*INFy*	*TGFβ*	*TNFα*
Main effect of protein content^2^											
NCP		1.72	1.52	2.00	1.24^a^	0.98	0.95	0.87^a^	1.08	0.90	0.83^a^
LCP		1.72	1.54	2.29	0.96^b^	0.81	0.91	0.64^b^	0.98	0.80	0.69^b^
Main effect of methionine content											
Met 2.8		1.43^bc^	1.51	1.62	1.19	0.95	1.18	0.80	1.21	0.85	0.76
Met 4.4		2.16^ a^	1.56	2.77	1.00	0.87	0.91	0.75	0.87	0.82	0.70
Met 6.0		1.25^c^	1.40	1.54	0.96	0.93	0.99	0.89	1.04	0.94	0.84
Met 7.6		1.75^abc^	1.42	2.42	1.28	0.73	0.70	0.60	0.96	0.74	0.66
Met 9.2		1.94^ab^	1.75	2.38	1.07	1.00	0.88	0.72	1.07	0.91	0.85
Interaction effect											
NCP	Met 2.8	1.46	1.54^ab^	1.43	1.40	0.98^ab^	1.01	0.97	1.33	0.85	0.82^ab^
NCP	Met 4.4	2.22	1.84^a^	3.03	1.06	0.86^ab^	0.91	0.85	0.87	0.80	0.59^b^
NCP	Met 6.0	1.00	1.00^b^	1.00	1.00	1.00^ab^	1.00	1.00	1.00	1.00	1.00^ab^
NCP	Met 7.6	1.76	1.35^ab^	2.45	1.28	0.66^b^	0.77	0.59	0.91	0.73	0.65^ab^
NCP	Met 9.2	2.01	1.86^a^	2.11	1.46	1.38^a^	1.07	0.94	1.26	1.12	1.09^a^
LCP	Met 2.8	1.41	1.49^ab^	1.81	0.98	0.92^ab^	1.36	0.64	1.08	0.85	0.70^ab^
LCP	Met 4.4	2.10	1.28^ab^	2.51	0.94	0.87^ab^	0.91	0.66	0.86	0.84	0.80^ab^
LCP	Met 6.0	1.50	1.80^a^	2.09	0.91	0.85^ab^	0.99	0.78	1.07	0.87	0.68^ab^
LCP	Met 7.6	1.74	1.48^ab^	2.39	1.28	0.80^ab^	0.62	0.60	1.01	0.76	0.67^ab^
LCP	Met 9.2	1.86	1.63^ab^	2.64	0.68	0.62^ab^	0.69	0.51	0.88	0.71	0.61^b^
*P*-value											
CP		0.815	0.851	0.423	0.011	0.072	0.751	0.023	0.514	0.101	0.034
Met		0.003	0.184	0.128	0.297	0.366	0.142	0.422	0.645	0.230	0.245
CP × Met		0.656	0.002	0.661	0.127	0.028	0.382	0.683	0.783	0.090	0.013
SEM		0.261	0.155	0.555	0.166	0.140	0.181	0.155	0.225	0.090	0.100

^a−c^Means within a column lacking a common superscript differ (*P* < 0.05)

^1^*IL1β* Interleukin 1 beta, *TNFα* Tumor necrotic factor alpha, *IFNγ* Interferon gamma, *IL10* Interleukin 10, *TGFβ* Transforming growth factor beta

^2^DPI, Day post inoculation; NCP, Normal protein group; LCP, Reduced protein group; Met, Methionine; Met 2.8, Dietary Met level = 2.8 g/kg; Met 4.4, Dietary Met level = 4.4 g/kg; Met 6.0, Dietary Met level = 6.0 g/kg; Met 7.6, Dietary Met level = 7.6 g/kg; Met 9.0, Dietary Met level = 9.0 g/kg

### Oxidative status

**Fig. 7:**
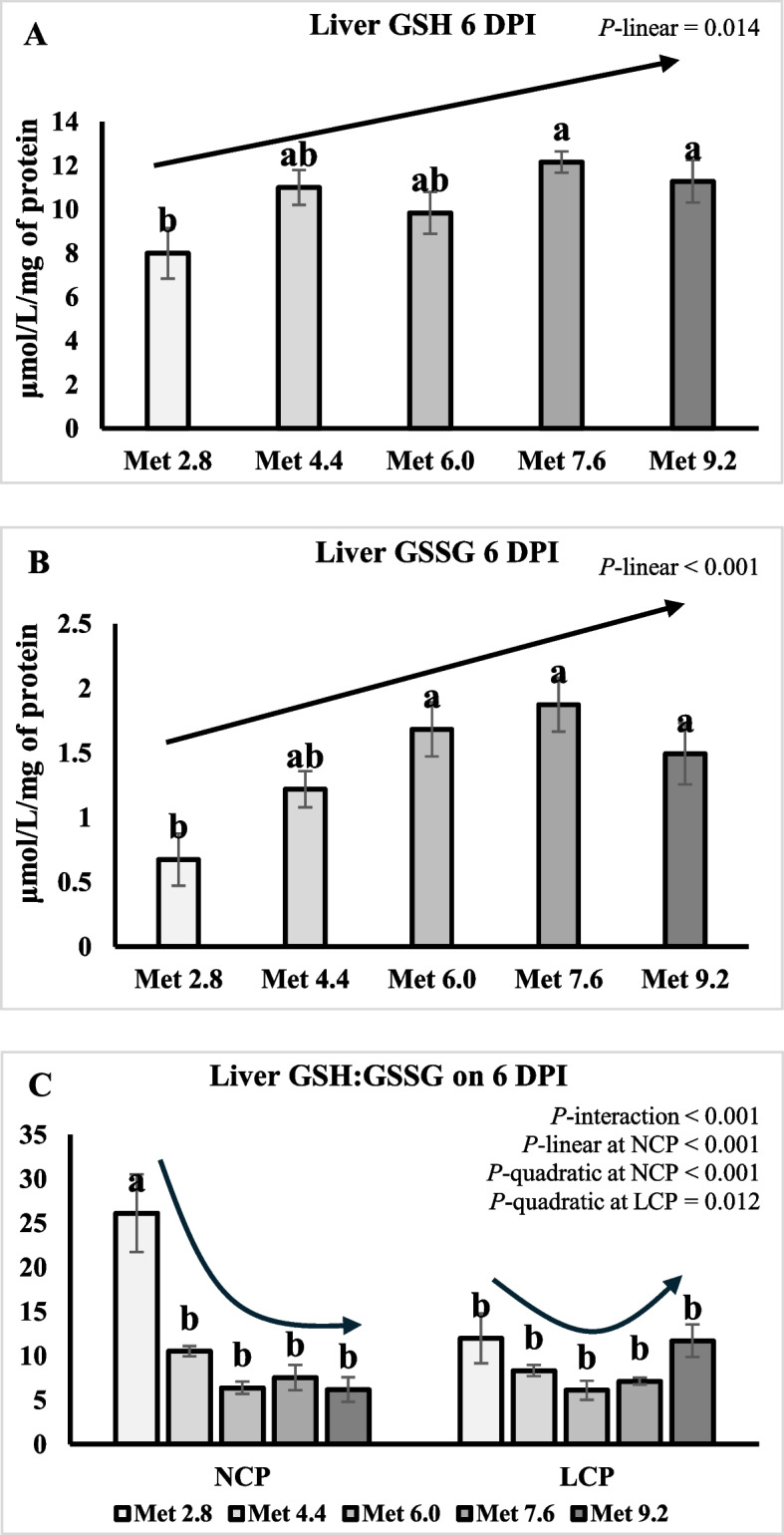
Effects of dietary methionine levels and protein contents on liver oxidative status of broilers challenged with *Eimeria* spp. on 6 DPI. The error bars represent the SEM values. Bars without a common letter differ significantly. The black lines with arrowhead represented significant linear or quadratic relationship between parameters and dietary methionine levels. Statistical significance was set at *P* ≤ 0.05. DPI, Day post inoculation; GSH, Glutathione; GSSH, Glutathione disulfide; Met, Methionine; NCP, Normal protein diet; LCP, Reduced protein diet; Met 2.8, Dietary Met level = 2.8 g/kg; Met 4.4, Dietary Met level = 4.4 g/kg; Met 6.0, Dietary Met level = 6.0 g/kg; Met 7.6, Dietary Met level = 7.6 g/kg; Met 9.0, Dietary Met level = 9.0 g/kg

**Fig. 8:**
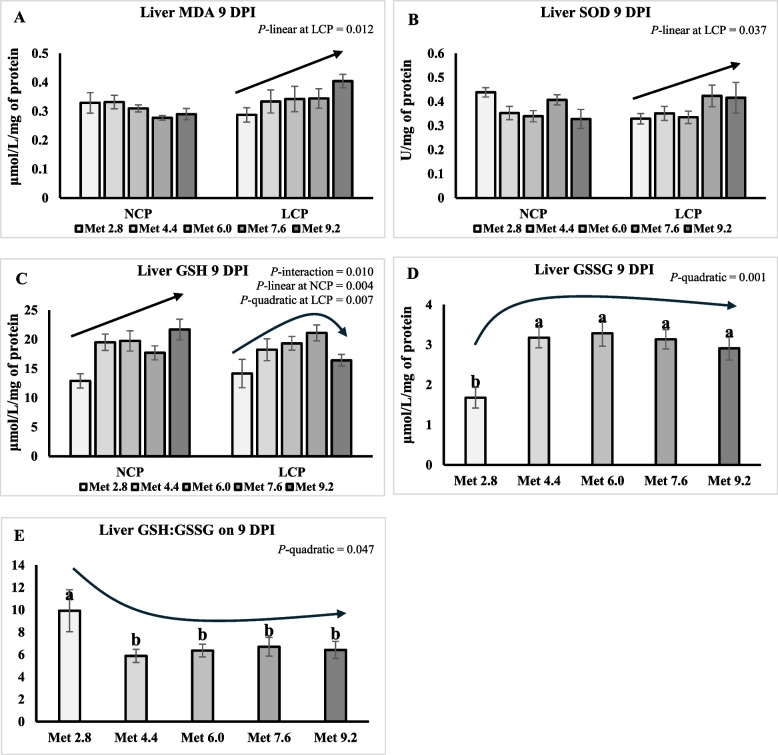
Effects of dietary methionine levels and protein contents on liver oxidative status of broilers challenged with *Eimeria* spp. on 9 DPI. The error bars represent the SEM values. Bars without a common letter differ significantly. The black lines with arrowhead represented significant linear or quadratic relationship between parameters and dietary methionine levels. Statistical significance was set at *P* ≤ 0.05. DPI, Day post inoculation; MDA, Malondialdehyde; SOD, Superoxide dismutase; GSH, Glutathione; GSSG, Glutathione disulfide; Met, Methionine; NCP, Normal protein diet; LCP, Reduced protein diet; Met 2.8, Dietary Met level = 2.8 g/kg; Met 4.4, Dietary Met level = 4.4 g/kg; Met 6.0, Dietary Met level = 6.0 g/kg; Met 7.6, Dietary Met level = 7.6 g/kg; Met 9.0, Dietary Met level = 9.0 g/kg

On 6 DPI, liver GSH was higher in the LCP groups than the NCP groups (*P* < 0.05) (Table 10). Liver GSH linearly increased as Met level increased (*P* < 0.01) (Fig. 7A). Liver GSSG quadratically increased as Met level increased (*P* < 0.01) (Fig. 7B). An interaction effect was observed for the GSH:GSSG ratio (*P* < 0.01), where the ratio quadratically decreased as Met level increased in the NCP groups, whereas the ratio exhibited a quadratic trend, decreasing initially and then increasing as Met level increased in the LCP groups (Fig. 7C). On 9 DPI, liver GSSG were significantly lower in the Met 2.8 groups compared to the other groups (*P* < 0.01) (Table 10). The GSH:GSSG ratio was significantly lower in the Met 4.4 groups compared to the Met 2.8 groups (*P* < 0.05). The MDA concentration and SOD activity linearly increased as Met level increased in the LCP groups (*P* < 0.05) (Fig. 8A and B). An interaction effect was observed for the GSH (*P* = 0.01), where GSH linearly increased as Met level increased in the NCP groups, whereas GSH exhibited a quadratic trend, increasing initially and then decreasing as Met level increased in the LCP groups (Fig. 8C). Liver GSSG quadratically increased whereas GSH:GSSG ratio quadratically decreased as Met level increased (*P* < 0.05) (Fig. 8D and E).

**Table 10:** Effects of dietary methionine levels and protein contents on liver oxidative status of broilers challenged with *Eimeria* spp.

Items^1^		6 DPI						9 DPI					
		MDA	SOD	GPX	GSH	GSSG	GSH/GSSG	MDA	SOD	GPX	GSH	GSSG	GSH/GSSG
Main effect of protein content^2^													
NCP		0.19	0.13	60.1	9.50^b^	1.26	11.3	0.31	0.37	46.6	18.3	2.69	7.36
LCP		0.19	0.13	61.8	11.2^a^	1.46	8.91	0.34	0.37	46.2	17.8	2.97	6.84
Main effect of methionine content													
Met 2.8		0.17	0.13	57.7	8.00	0.675^b^	19.0^a^	0.31	0.38	47.2	13.5^b^	1.66^b^	10.1^a^
Met 4.4		0.19	0.13	62.5	11.3	1.22^ab^	9.56^b^	0.33	0.35	45.9	18.9^a^	3.16^a^	5.88^b^
Met 6.0		0.17	0.14	62.6	9.79	1.55^a^	6.25^b^	0.33	0.34	45.4	19.5^a^	3.28^a^	6.35^ab^
Met 7.6		0.22	0.14	59.1	11.5	1.87^a^	6.86^b^	0.31	0.41	49.2	19.4^a^	3.13^a^	6.69^ab^
Met 9.2		0.19	0.13	62.8	11.3	1.49^a^	8.94^b^	0.35	0.37	44.4	19.0^a^	2.91^a^	6.49^ab^
Interaction effect													
NCP	Met 2.8	0.18	0.13	58.9	5.46	0.239	26.1^a^	0.33	0.44	47.0	12.9	1.48	11.5
NCP	Met 4.4	0.18	0.13	60.7	11.0	1.07	10.5^b^	0.33	0.35	48.1	19.5	3.04	5.99
NCP	Met 6.0	0.14	0.13	60.0	9.93	1.42	6.39^b^	0.31	0.34	45.9	19.7	2.96	6.71
NCP	Met 7.6	0.24	0.14	60.3	11.9	1.87	7.54^b^	0.28	0.41	48.3	17.7	3.39	5.32
NCP	Met 9.2	0.19	0.12	60.3	9.22	1.70	6.18^b^	0.29	0.33	43.9	21.7	2.58	7.24
LCP	Met 2.8	0.16	0.13	56.4	10.5	1.11	12.0^b^	0.29	0.33	47.4	14.2	1.83	8.63
LCP	Met 4.4	0.20	0.12	64.4	11.6	1.37	8.61^b^	0.33	0.35	43.8	18.2	3.29	5.77
LCP	Met 6.0	0.20	0.14	65.1	9.64	1.69	6.11^b^	0.34	0.33	44.9	19.3	3.61	5.99
LCP	Met 7.6	0.20	0.14	57.9	11.1	1.87	6.17^b^	0.34	0.42	50.0	21.1	2.88	8.06
LCP	Met 9.2	0.20	0.13	65.3	13.3	1.28	11.7^b^	0.40	0.42	44.9	16.4	3.24	5.75
*P*-value													
CP		0.909	0.836	0.421	0.033	0.268	0.060	0.069	0.916	0.752	0.651	0.278	0.572
Met		0.741	0.590	0.444	0.035	0.002	< 0.001	0.673	0.215	0.279	0.003	0.001	0.048
CP × Met		0.713	0.748	0.635	0.074	0.262	< 0.001	0.107	0.119	0.705	0.010	0.560	0.394
SEM		0.040	0.009	3.45	1.24	0.285	1.99	0.029	0.034	2.27	1.59	0.397	1.44

^a,b^Means within a column lacking a common superscript differ (*P* < 0.05)

^1^MDA, Malondialdehyde (μmol/L/mg of protein); SOD, superoxide dismutase (U/mg of protein); GPX, glutathione peroxidase (nmol/min/mg of protein); GSH, glutathione (μmol/L/mg of protein); GSSG, glutathione disulfide (μmol/L/mg of protein)

^2^DPI, Day post inoculation; NCP, Normal protein group; LCP, Reduced protein group; Met, Methionine; Met 2.8, Dietary Met level = 2.8 g/kg; Met 4.4, Dietary Met level = 4.4 g/kg; Met 6.0, Dietary Met level = 6.0 g/kg; Met 7.6, Dietary Met level = 7.6 g/kg; Met 9.0, Dietary Met level = 9.0 g/kg

### Oocyst shedding

**Fig. 9:**
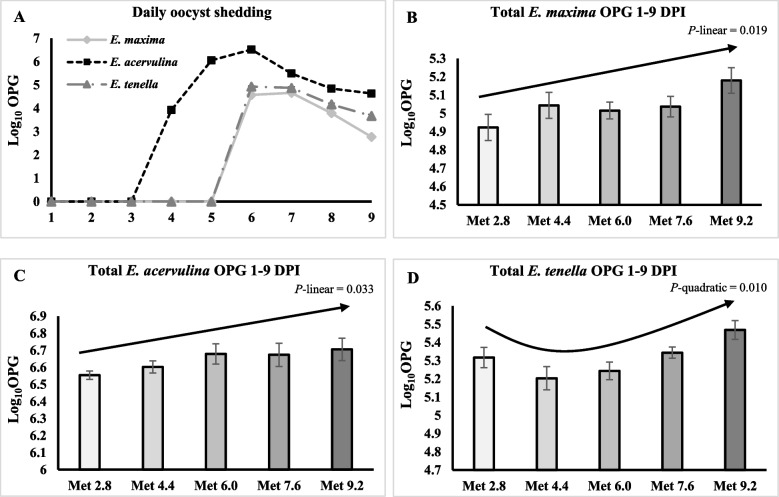
Effects of dietary methionine levels and protein contents on oocyst shedding of broilers challenged with *Eimeria* spp. The results were expressed as the log_10_ of oocysts per gram of excreta. The error bars represent the SEM values. The black lines with arrowhead represented significant linear or quadratic relationship between parameters and dietary methionine levels. Statistical significance was set at *P* ≤ 0.05. OPG, Oocyst per gram of excreta; DPI, Day post inoculation; Met, Methionine; NCP, Normal protein diet; LCP, Reduced protein diet; Met 2.8, Dietary Met level = 2.8 g/kg; Met 4.4, Dietary Met level = 4.4 g/kg; Met 6.0, Dietary Met level = 6.0 g/kg; Met 7.6, Dietary Met level = 7.6 g/kg; Met 9.0, Dietary Met level = 9.0 g/kg

The oocysts of *E. acervulina* were first detected in the excreta on 3 DPI, and the oocysts of *E. maxima* and *E. tenella* first appeared in the excreta on 5 DPI. The OPG of three species peaked at 6 DPI (Fig. 9A). The accumulated OPG from 1 to 9 DPI of *E. tenella* was significantly lower in the Met 4.4 and Met 6.0 groups than the Met 9.2 groups (Table 11). The accumulated OPG from 1 to 9 DPI of *E. acervulina* and *E. maxima* linearly increased as Met level increased (*P* < 0.05) (Fig. 9B and 9C). The accumulated OPG from 1 to 9 DPI of *E. tenella* changed quadratically as Met level increased (*P* < 0.05) (Fig. 9D).

**Table 11:** Effects of dietary methionine levels and protein contents on total oocyst shedding from 0 to 9 day post inoculation of broilers under *Eimeria* challenge

Items^1^		*E. acervulina*^2^	*E. maxima*	*E. tenella*
Main effect of protein content				
NCP		6.66	5.01	5.31
LCP		6.27	5.07	5.32
Main effect of methionine content				
Met 2.8		6.55	4.92	5.32^ab^
Met 4.4		6.60	5.04	5.20^b^
Met 6.0		6.68	5.03	5.24^b^
Met 7.6		6.67	5.04	5.34^ab^
Met 9.2		6.71	5.18	5.47^a^
Interaction effect				
NCP	Met 2.8	6.53	4.94	5.25
NCP	Met 4.4	6.54	5.01	5.21
NCP	Met 6.0	6.66	4.94	5.26
NCP	Met 7.6	6.75	4.98	5.39
NCP	Met 9.2	6.65	5.18	5.44
LCP	Met 2.8	6.57	4.91	5.38
LCP	Met 4.4	6.66	5.07	5.19
LCP	Met 6.0	6.70	5.11	5.23
LCP	Met 7.6	6.59	5.10	5.30
LCP	Met 9.2	6.76	5.17	5.49
*P*-value				
CP		0.513	0.285	0.839
Met		0.262	0.124	0.015
CP × Met		0.369	0.802	0.652
SEM		0.068	0.094	0.079

^a,b^Means within a column lacking a common superscript differ (*P* < 0.05)

^1^DPI, Days post inoculation; NCP, Normal protein group; LCP, Reduced protein group; Met, Methionine; Met 2.8, Dietary Met level = 2.8 g/kg; Met 4.4, Dietary Met level = 4.4 g/kg; Met 6.0, Dietary Met level = 6.0 g/kg; Met 7.6, Dietary Met level = 7.6 g/kg; Met 9.0, Dietary Met level = 9.0 g/kg

^2^The results were expressed as the log_10_ of oocysts per gram of excreta

### Gene expression of methionine and folate metabolism enzymes

**Fig. 10:**
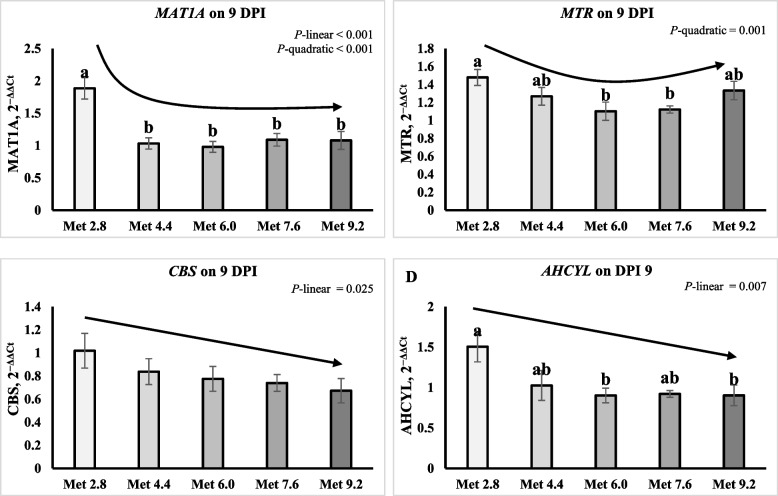
Effects of dietary methionine levels and protein contents on expression of methionine metabolism enzymes of broilers challenged with *Eimeria* spp. The error bars represent the SEM values. Bars without a common letter differ significantly. The black lines with arrowhead represented significant linear or quadratic relationship between parameters and dietary methionine levels. Statistical significance was set at *P* ≤ 0.05. DPI, Day post inoculation; *MAT1A*, Methionine adenosyltransferase 1A; *MTR*, Methionine synthase; *CBS*, Cystathionine beta synthase; *AHCYL*, Adenosylhomocysteinase like 1; Met, Methionine; NCP, Normal protein diet; LCP, Reduced protein diet; Met 2.8, Dietary Met level = 2.8 g/kg; Met 4.4, Dietary Met level = 4.4 g/kg; Met 6.0, Dietary Met level = 6.0 g/kg; Met 7.6, Dietary Met level = 7.6 g/kg; Met 9.0, Dietary Met level = 9.0 g/kg

No significant effects were observed for expression of Met and folate metabolism enzymes on 6 DPI (Table 12). On 9 DPI, the expression of methionine adenosyltransferase 1A (*MAT1A*), methionine synthase (*MTR*), and cystathionine beta synthase (*CBS*) were lower in the LCP groups than in the NCP groups (*P* < 0.05). The expression of *MAT1A* was significantly higher in the Met 2.8 groups than the other groups (*P* < 0.01). The expression of adenosylhomocysteinase like 1 (*AHCYL1*) was higher in the Met 2.8 groups than the Met 6.0 and Met 9.2 groups (*P* < 0.05). The expression of *CBS* and *AHCYL1* linearly decreased and the expression of *MAT1A* and *MTR* quadratically decreased as Met level decreased (*P* < 0.05) (Fig. 10A–D).

**Table 12:** Effects of dietary methionine levels and protein contents on expression of methionine metabolism enzymes of broilers challenged with *Eimeria* spp.

Items^1^		6 DPI				9 DPI			
		*MAT1A*	*MTR*	*AHCYL1*	*CBS*	*MAT1A*	*MTR*	*AHCYL1*	*CBS*
Main effect of protein content^2^									
NCP		0.87	1.03	0.79	0.74	1.52^a^	1.16^a^	1.16	0.97^a^
LCP		0.85	1.10	0.79	0.70	1.18^b^	0.99^b^	0.99	0.67^b^
Main effect of methionine content									
Met 2.8		0.90	1.28	0.71	0.78	2.05^a^	1.26^a^	1.51^a^	1.02
Met 4.4		0.78	1.07	0.69	0.61	1.28^b^	1.05^ab^	1.03^ab^	0.84
Met 6.0		0.85	0.95	0.85	0.84	0.98^b^	1.00^ab^	0.90^b^	0.80
Met 7.6		0.90	1.03	0.86	0.70	1.35^b^	0.94^b^	1.02^ab^	0.74
Met 9.2		0.88	1.01	0.84	0.65	1.08^b^	1.12^ab^	0.90^b^	0.69
Interaction effect									
NCP	Met 2.8	0.83	1.15	0.57	0.71	2.47	1.41	1.59	1.28
NCP	Met 4.4	0.86	1.03	0.86	0.66	1.44	1.17	1.04	0.81
NCP	Met 6.0	1.00	1.00	1.00	1.00	1.00	1.00	1.00	1.00
NCP	Met 7.6	0.74	1.05	0.66	0.67	1.48	0.96	1.15	0.85
NCP	Met 9.2	0.94	0.93	0.86	0.65	1.23	1.27	1.00	0.90
LCP	Met 2.8	0.97	1.42	0.86	0.85	1.63	1.11	1.43	0.75
LCP	Met 4.4	0.69	1.10	0.53	0.57	1.12	0.93	1.01	0.87
LCP	Met 6.0	0.70	0.89	0.71	0.67	0.96	1.00	0.80	0.60
LCP	Met 7.6	1.06	1.00	1.05	0.73	1.23	0.93	0.89	0.63
LCP	Met 9.2	0.82	1.09	0.82	0.65	0.93	0.98	0.81	0.49
*P*-value									
CP		0.807	0.476	0.969	0.556	0.023	0.014	0.208	0.002
Met		0.942	0.203	0.719	0.269	< 0.001	0.055	0.041	0.206
CP × Met		0.388	0.714	0.095	0.288	0.533	0.459	0.987	0.272
SEM		0.169	0.147	0.173	0.114	0.232	0.108	0.209	0.143

^a,b^Means within a column lacking a common superscript differ (*P* < 0.05)

^1^*MAT1A* Methionine adenosyltransferase 1A, *AHCYL1* Adenosylhomocysteinase like 1, *MTR* Methionine synthase, *CBS* Cystathionine beta synthase

^2^DPI, Day post inoculation; NCP, Normal protein group; LCP, Reduced protein group; Met, Methionine; Met 2.8, Dietary Met level = 2.8 g/kg; Met 4.4, Dietary Met level = 4.4 g/kg; Met 6.0, Dietary Met level = 6.0 g/kg; Met 7.6, Dietary Met level = 7.6 g/kg; Met 9.0, Dietary Met level = 9.0 g/kg

**Fig. 11:**
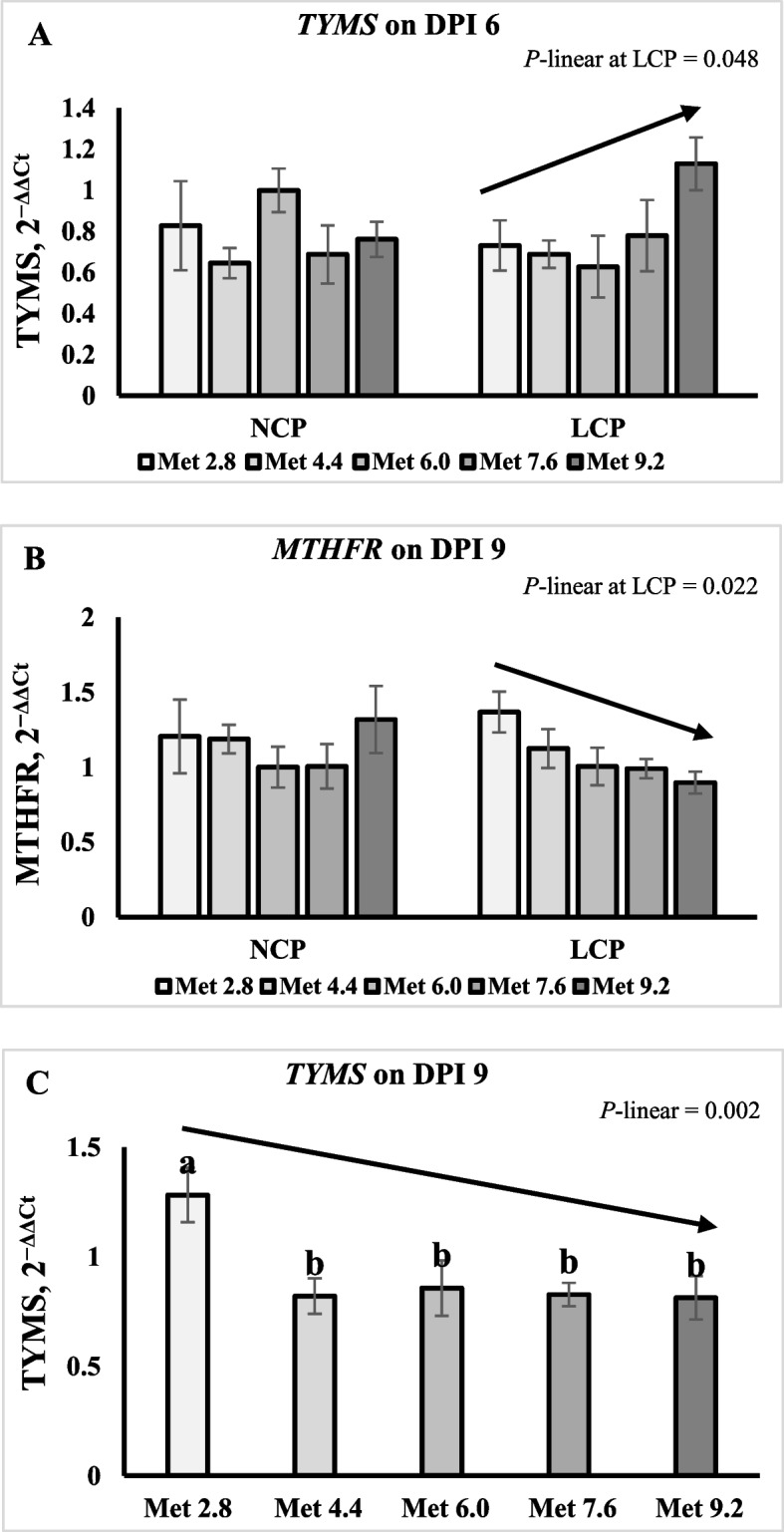
Effects of dietary methionine levels and protein contents on expression of folate metabolism enzymes of broilers challenged with *Eimeria* spp. The error bars represent the SEM values. Bars without a common letter differ significantly. The black lines with arrowhead represented significant linear or quadratic relationship between parameters and dietary methionine levels. Statistical significance was set at *P* ≤ 0.05. DPI, Day post inoculation; *TYMS*, Thymidylate synthase; *DHFR*, Dihydrofolate reductase; *MTHFD1*, Methylenetetrahydrofolate dehydrogenase 1; *MTHFR*, Methylenetetrahydrofolate reductase; Met, Methionine; NCP, Normal protein diet; LCP, Reduced protein diet; Met 2.8, Dietary Met level = 2.8 g/kg; Met 4.4, Dietary Met level = 4.4 g/kg; Met 6.0, Dietary Met level = 6.0 g/kg; Met 7.6, Dietary Met level = 7.6 g/kg; Met 9.0, Dietary Met level = 9.0 g/kg

On 6 DPI, the expression of thymidylate synthase (*TYMS*) in the LCP groups linearly increased as Met level increased (*P* < 0.05) (Fig. 11A). On 9 DPI, the expression of dihydrofolate reductase (*DHFR*) and methylenetetrahydrofolate dehydrogenase 1 (*MTHFD1*) were lower in the LCP groups than in the NCP groups (*P* < 0.01) (Table 13). The expression of *TYMS* was significantly higher in the Met 2.8 groups than the other groups (*P* < 0.01). The expression of methylenetetrahydrofolate reductase (*MTHFR*) in the LCP groups and *TYMS* linearly decreased as Met level increased (*P* < 0.05) Fig. 11B and C.

**Table 13:** Effects of dietary methionine levels and protein contents on expression of folate metabolism enzymes of broilers challenged with *Eimeria* spp.

Items^1^		6 DPI					9 DPI				
		*MTHFR*	*DHFR*	*SHMT*	*MTHFD1*	*TYMS*	*MTHFR*	*DHFR*	*SHMT*	*MTHFD1*	*TYMS*
Main effect of protein content^2^											
NCP		1.00	0.81	0.82	0.75	0.71	1.14	1.00^a^	0.99	0.94^a^	1.03
LCP		1.14	0.84	0.99	0.82	0.73	1.08	0.68^b^	0.80	0.66^b^	0.86
Main effect of methionine content											
Met 2.8		1.07	0.82	0.84	0.83	0.77	1.29	0.85	0.90	0.91	1.40^a^
Met 4.4		1.12	0.70	0.77	0.61	0.59	1.16	0.85	0.91	0.78	0.82^b^
Met 6.0		1.02	0.85	0.95	0.90	0.78	1.00	0.84	0.92	0.82	0.86^b^
Met 7.6		1.10	0.91	1.00	0.86	0.65	1.00	0.68	0.74	0.73	0.83^b^
Met 9.2		1.02	0.83	0.96	0.74	0.83	1.11	0.96	1.00	0.76	0.81^b^
Interaction effect											
NCP	Met 2.8	0.99	0.72	0.73	0.75	0.73	1.21	0.88	1.03	1.03	1.46
NCP	Met 4.4	0.99	0.73	0.81	0.63	0.57	1.19	1.03	0.93	0.84	0.78
NCP	Met 6.0	1.00	1.00	1.00	1.00	1.00	1.00	1.00	1.00	1.00	1.00
NCP	Met 7.6	0.99	0.79	0.67	0.59	0.61	1.01	0.73	0.77	0.85	0.92
NCP	Met 9.2	1.03	0.82	0.92	0.77	0.67	1.32	1.35	1.21	0.96	0.97
LCP	Met 2.8	1.15	0.93	0.96	0.91	0.80	1.37	0.83	0.78	0.80	1.35
LCP	Met 4.4	1.26	0.66	0.73	0.58	0.61	1.12	0.66	0.89	0.73	0.86
LCP	Met 6.0	1.05	0.70	0.91	0.80	0.55	1.01	0.68	0.84	0.64	0.71
LCP	Met 7.6	1.22	1.04	1.34	1.13	0.69	0.99	0.63	0.71	0.60	0.74
LCP	Met 9.2	1.02	0.85	1.01	0.71	0.99	0.90	0.58	0.80	0.56	0.65
*P*-value											
CP		0.065	0.768	0.200	0.402	0.872	0.476	0.002	0.054	0.001	0.115
Met		0.866	0.609	0.746	0.249	0.334	0.282	0.457	0.464	0.575	0.002
CP × Met		0.704	0.266	0.331	0.102	0.089	0.367	0.156	0.698	0.664	0.740
SEM		0.117	0.135	0.198	0.140	0.133	0.146	0.152	0.145	0.137	0.159

^a,b^Means within a column lacking a common superscript differ (*P* < 0.05)

^1^*MTHFR* Methylenetetrahydrofolate reductase, *DHFR* Dihydrofolate reductase, *SHMT* Serine hydroxymethyltransferase, *MTHFD1* Methylenetetrahydrofolate dehydrogenase 1, *TYMS* Thymidylate synthase

^2^DPI, Day post inoculation; NCP, Normal protein group; LCP, Reduced protein group; Met, Methionine; Met 2.8, Dietary Met level = 2.8 g/kg; Met 4.4, Dietary Met level = 4.4 g/kg; Met 6.0, Dietary Met level = 6.0 g/kg; Met 7.6, Dietary Met level = 7.6 g/kg; Met 9.0, Dietary Met level = 9.0 g/kg

## Discussion

The present study investigated the impact of dietary Met levels and protein content on growth performance, intestinal health, oxidative status, and gene expression related to methionine and folate metabolism enzymes in broiler chickens. The multifaceted evaluation of these parameters provided a comprehensive understanding of how Met levels and low dietary protein affect the performance and health of broilers under *Eimeria* challenge.

The daily FI began to decrease on 4 DPI and reached its lowest point on 6 DPI. This decrease in daily FI in response to *Eimeria* challenge aligned with the reproductive cycle of the parasites. It has been shown that *Eimeria* spp. typically undergo the asexual reproduction (schizogony) to produce merozoites from 3 to 5 DPI, the merozoites subsequently re-enter the enterocytes to initiate sexual reproduction (gametogony) around 6 to 7 DPI, resulting in severe damage to the intestinal mucosa [6, 53]. This damage to the intestinal mucosa and integrity may cause pain and discomfort in the birds, potentially leading to anorexia [54]. After 6 DPI, the birds gradually recovered from the infection, and the FI increased accordingly. The same pattern has been reported by previous studies wherein broilers were also subjected to *Eimeria* challenge, and the daily change in FI was monitored [3, 4, 6, 10].

In the present study, we observed that in the first three DPI, the FI linearly or quadratically increased as Met levels increased from deficiency to levels higher than recommendation in the NCP groups. According to studies conducted by previous researchers who fed broilers with diets containing graded levels of Met, Met deficiency could lead to suppression in FI, which be restored by increasing Met inclusion in diet [21, 55, 56], as observed in this current study in the NCP groups. However, the birds did not respond significantly to the changes of dietary Met levels in the LCP groups, this observation suggested that the birds’ response to dietary Met levels might be dampened by the decreased CP content in the diet. To the authors’ knowledge, this discrepancy in the response of FI to dietary Met levels across different levels of CP diet has not been previously reported. Further studies are warranted to elucidate the underlying mechanism behind this difference. More intriguingly, starting from 4 DPI corresponding with the reproduction of *Eimeria* spp. and the subsequent drop in FI, birds in the higher Met groups began to exhibit lower FI, especially in the LCP groups. By 6 DPI, when the infection was most acute, FI linearly decreased as Met levels increased. This observation suggested that during the acute infection of coccidiosis, higher dietary Met levels might exert adverse effects on the birds, potentially contributing to the promotion of *Eimeria* reproduction. While only daily FI was observed in the current study, it would be interesting for future research to monitor daily BW changes to further support this hypothesis. The varying patterns in the birds’ FI response to dietary Met levels on each DPI as *Eimeria* infection progressed could be attributed to certain physiological changes induced by the infection. Further investigation is necessary to elucidate the exact mechanism behind this change.

Nevertheless, for overall growth performance indices, increased Met levels linearly improved the BWG and FCR from 0 to 6 DPI. As the first limiting amino acid in the poultry diet [14], Met deficiency has been shown to hindered the growth performance of broilers raised under unchallenged conditions [21, 57, 58], this current research provided further evidence underscoring the crucial role of Met in sustaining the growth performance of the birds under *Eimeria* challenge. However, it is worth noting that although a linear trend between Met levels and performance indices were observed, this improvement in growth performance seemed to plateau when the Met levels reached 6.0 g/kg especially from 7 to 9 DPI where a quadratic trend was observed between FCR and Met levels. This observation suggested that the higher Met supplementation beyond this level might not necessarily lead to improvement in the growth performance for birds under *Eimeria* challenge. Interestingly, despite previous research showed that reduced CP levels in diets could compromise the performance of the birds [59, 60], this decrease in growth performance caused by lower dietary CP was not observed in the current study. While the previous studies were conducted in unchallenged birds, the anorexia induced by coccidiosis and accompanying physiological changes in the current study might lead to a less prominent compromising effect of reduced CP levels on growth performance. This observation aligns with findings by Teng et al. [38], who also fed broilers with reduced CP diets under *Eimeria* challenge.

For the influence of Met levels and protein contents on intestinal morphology, we again observed that the protein contents affected the duodenal VH:CD ratios differently on two timepoints. On 6 DPI, the LCP groups had higher VH:CD than the NCP groups, while the opposite pattern was observed on 9 DPI. Similarly results have been reported by previous studies finding that reduced dietary protein resulted in higher duodenal VH:CD ratio in the acute infection phase while not in the recovery phase [38]. In the acute infection phase, a reduction in dietary protein content could plausibly diminish the trypsin secretion, a factor responsible for the excystation of sporozoites from sporocysts [38, 61, 62]. This attenuation may result in an elevated VH:CD ratio which was generally considered as an indicator for better morphology and absorption function [63–65]. Conversely, during the recovery phase, the protein deficiency might have impeded the recovery processes, contributing to the lower VH:CD ratio in the LCP groups. A previous swine study has reported that the reduced protein diet negatively impacted the duodenal morphology [66]. Regarding the quadratic trends observed in jejunal CD and duodenal VH:CD ratio with increasing Met levels in the current study, it suggested that both low and high Met levels might confer benefits to the intestinal morphology of broilers under coccidia challenge. Given the close association of Met with folate metabolism and the synthesis of SAM [37, 67], and considering the significance of both compounds for parasite replication [25, 39, 40], lower Met levels could potentially lead to reduced *Eimeria* reproduction and lessened damage to the intestinal morphology by reducing the metabolism of folate and synthesis of SAM. On the other hand, the higher Met levels could mitigate the damage caused by infection due to the importance of Met in protein synthesis and its antioxidant capacity [14, 15, 68]. Further research needs to confirm the above hypothesis and investigate the mechanism behind the observation.

The increased Met levels exhibited contrasting effects on the gene expression of tight junction proteins at both time points in the current study. On 6 DPI, increased Met levels linearly increased expression of *CLDN1* and *ZO2* in the jejunum, aligning with findings from prior studies where increased Met supplementation also elevated the tight junction protein expression in broilers [20, 23]. This underscores the importance of Met in preserving intestinal integrity; however, intriguingly, on 9 DPI, the linear increase in Met was associated with a decrease in the expression of these genes in the NCP groups. This provides additional evidence that birds underwent physiological changes during *Eimeria* infection, leading to the varied responses to dietary factors on different timepoints. As for the gene expression of amino acid transporters, lower Met levels were associated with higher expression of these genes, similar results have also been reported by Fagundes et al. [69] who fed the broilers diets with two different levels of Met. This observation suggested that the birds may have an alternative compensatory mechanism to address Met deficiency, distinct from merely increasing their FI. Increased Met levels quadratically affected the gene expression of cytokines in the NCP groups, a similar quadratic trend was reported in a previous research where the birds were fed diets containing graded levels of Met with normal CP content [21]. However, such trend was not observed in the LCP groups, indicating a potential modulation in the regulatory pathways associated with cytokine gene expression and Met levels in the context of varying dietary protein contents. It is noteworthy that reducing the protein content led to a decrease in the gene expression of cytokines, underscoring the crucial role of protein in affecting certain aspects of immune responses of broilers as highlighted in serval previous review papers discussing the importance of protein or essential amino acids in regulating immune responses [11, 70–72].

The current study provided further evidence for the importance of Met in the production of GSH, the important endogenous antioxidants [26, 73, 74], as the GSH and GSSG contents linearly increased as Met level increased. This observation provided some support to the above proposed hypothesis that the higher Met level mitigated the damage of *Eimeria* infection to the intestinal morphology by alleviating the infection-induced oxidative stress. While the essential role of Met in GSH synthesis has been documented in other studies [20, 75, 76], the results from the current study also suggested that the improvement of GSH or GSSG profiles by increasing Met levels seemed to be less prominent on 9 DPI when multiple previous studies have consistently demonstrated the oxidative stress caused by coccidiosis diminished as the birds recovered from the infection [4, 20, 21, 77]. These findings collectively underscored the essential relationship among Met, GSH production, and the birds’ dynamic response to *Eimeria* infection over time.

The daily oocyst shedding trend corresponded to the parasite reproduction cycle, and the similar trend has been reported by previous research investigating the oocyst shedding in broilers infected with mixed species of *Eimeria* [78, 79]. The current study unveiled a linear or quadratic increase in oocyst shedding across all three species in response to elevated Met levels. This outcome substantiated the hypothesis that higher Met levels could promote the reproduction of *Eimeria*. A previous study has also reported that additional Met supplementation increased oocyst shedding in *Eimeria*-challenged broilers [38]. While the lesion score was not assessed in the present study, since Met primarily impacts antioxidant capacity and immune regulation, observing marked differences in lesion scores may be less likely. Nevertheless, in the future study, incorporating this assessment should be considered. To explore the potential mechanisms behind this increase in oocyst shedding, we examined whether the increase in Met level upregulated the expression of key enzymes associated with folate and SAM metabolism as previous studies have highlighted their intricate connection and essential roles in DNA synthesis and methylation for parasites [6, 19, 23, 24]. The results showed that the increased Met level only linearly increased the expression of *TYMS* in the LCP groups on 6 DPI, which is the key enzyme regulating the production of dTMP required for DNA synthesis and repair [80, 81]. More intriguingly, on 9 DPI the increased Met linearly or quadratically decreased the expression of these key enzymes. In other words, the birds increased the expression of these enzymes, possibly to compensate for the deficiency in dietary Met levels. Further studies may need to delve deeper into the analysis of the actual protein levels or activities of these enzymes to better understand how Met level may affect the metabolism of folate and SAM and in turn affect the reproduction of *Eimeria*. Additionally, the results showed that expression of these enzymes was lower in the LCP groups than the NCP groups, suggesting an existed interplay between protein content and metabolism of other nutrients.

## Conclusion

In summary, in broilers challenged with *Eimeria* spp., although increasing Met levels improved the growth performance of the birds from 0 to 6 DPI, such improvement tended to plateau with the Met level reaching 6.0 g/kg. Interestingly, reducing the dietary CP did not deteriorate the growth performance of the birds. The dietary CP contents and Met levels exerted contrasting effects on intestinal morphology and tight junction protein expression on 6 and 9 DPI, whereas increasing Met consistently decreased the expression of amino acid transporters. Significant quadratic relationships were observed between Met levels and liver GSH concentrations, underscoring the role of Met in the antioxidant system. It was worth noting that the increasing Met levels led to increased oocyst shedding which might be related to the role of Met in folate metabolism. In conclusion, our results indicated that reducing the dietary CP level by 3% with 6.0 g/kg of Met could maintain the performance and intestinal health of broilers under *Eimeria* challenge. Future study could seek to investigate the mechanism behind the increased oocyst shedding observed in this current study and its influences for subsequent flock performance on the same litter.

## Data Availability

All data from this study are available from the corresponding author upon reasonable request.

## Abbreviations

*AHCYL1*: Adenosylhomocysteinase like 1
BW: Body weight
BWG: Body weight gain
*CBS*: Cystathionine beta synthase
CD: Crypt depth
*CLDN1*: Claudin 1
CP: Crude protein
CT: Cecal tonsil
*DHFR*: Dihydrofolate reductase
DPI: Day post inoculation
FCR: Feed conversion ratio
FI: Feed intake
GPX: Glutathione peroxidase
GSSG: Glutathione disulfide
GSH: Glutathione
*IFNγ*: Interferon gamma
*IL1β*: Interleukin 1 beta
*IL10*: Interleukin 10
LCP: Reduced protein diet
*MAT1A*: Methionine adenosyltransferase 1A
MDA: Malondialdehyde
Met: Methionine
*MTHFD1*: Methylenetetrahydrofolate dehydrogenase 1
*MTHFR*: Methylenetetrahydrofolate reductase
*MTR*: Methionine synthase
*MUC2*: Mucin 2
NCP: Normal protein diet
*OCLN*: Occludin
OPG: Oocyst per gram of excreta
SAM: *S*-adenosylmethionine
*SHMT*: Serine hydroxymethyltransferase
*SLC6A19*: Sodium-dependent neutral amino acid transporter B(0)AT1
*SLC7A9*: B(0, +)-type amino acid transporter 1
*SLC43A2*: L-type amino acid transporter 4
SOD: Superoxide dismutase
*TGFβ*: Transforming growth factor beta
*TNFα*: Tumor necrotic factor alpha
*TYMS*: Thymidylate synthase
VH: Villus height
*ZO2*: Zonula occludens 2

## References

[1] Blake DP, Knox J, Dehaeck B, Huntington B, Rathinam T, Ravipati V (2020). Re-calculating the cost of coccidiosis in chickens. Vet Res.

[2] Mesa-Pineda C, Navarro-Ruíz JL, López-Osorio S, Chaparro-Gutiérrez JJ, Gómez-Osorio LM (2021). Chicken coccidiosis: From the parasite lifecycle to control of the disease. Front Vet Sci.

[3] Choi J, Goo D, Sharma MK, Ko H, Liu G, Paneru D (2023). Effects of different *Eimeria* inoculation doses on growth performance, daily feed intake, gut health, gut microbiota, foot pad dermatitis, and *Eimeria* gene expression in broilers raised in floor pens for 35 days. Animals.

[4] Sharma MK, Liu G, White DL, Tompkins YH, Kim WK (2022). Effects of mixed *Eimeria* challenge on performance, body composition, intestinal health, and expression of nutrient transporter genes of Hy-Line W-36 pullets (0–6 wks of age). Poult Sci..

[5] Teng P-Y, Yadav S, Castro FLdS, Tompkins YH, Fuller AL, Kim WK (2020). Graded *Eimeria* challenge linearly regulated growth performance, dynamic change of gastrointestinal permeability, apparent ileal digestibility, intestinal morphology, and tight junctions of broiler chickens. Poult Sci.

[6] Sharma MK, Liu G, White DL, Kim WK (2024). Graded levels of *Eimeria* infection linearly reduced the growth performance, altered the intestinal health, and delayed the onset of egg production of Hy-Line W-36 laying hens when infected at the prelay stage. Poult Sci.

[7] Abbas RZ, Iqbal Z, Blake D, Khan MN, Saleemi MK (2011). Anticoccidial drug resistance in fowl coccidia: the state of play revisited. J World's Poult Sci.

[8] Chapman HD, Roberts B, Shirley MW, Williams RB (2005). Guidelines for evaluating the efficacy and safety of live anticoccidial vaccines, and obtaining approval for their use in chickens and turkeys. Avian Pathol.

[9] Chapman HD, Cherry TE, Danforth HD, Richards G, Shirley MW, Williams RB (2002). Sustainable coccidiosis control in poultry production: the role of live vaccines. Int J Parasitol.

[10] Goo D, Choi J, Ko H, Choppa VSR, Liu G, Lillehoj HS (2023). Effects of *Eimeria maxima* infection doses on growth performance and gut health in dual-infection model of necrotic enteritis in broiler chickens. Front Physiol..

[11] Tourkochristou E, Triantos C, Mouzaki A (2021). The influence of nutritional factors on immunological outcomes. Front Immunol.

[12] Adedokun SA, Olojede OC (2018). Optimizing gastrointestinal integrity in poultry: The role of nutrients and feed additives. Front Vet Sci.

[13] Liu G, Ajao AM, Shanmugasundaram R, Taylor J, Ball E, Applegate TJ (2023). The effects of arginine and branched-chain amino acid supplementation to reduced-protein diet on intestinal health, cecal short-chain fatty acid profiles, and immune response in broiler chickens challenged with *Eimeria* spp. Poult Sci.

[14] Liu G, Magnuson AD, Sun T, Tolba SA, Starkey C, Whelan R (2019). Supplemental methionine exerted chemical form-dependent effects on antioxidant status, inflammation-related gene expression, and fatty acid profiles of broiler chicks raised at high ambient temperature. J Anim Sci.

[15] Magnuson AD, Liu G, Sun T, Tolba SA, Xi L, Whelan R, et al. Supplemental methionine and stocking density affect antioxidant status, fatty acid profiles, and growth performance of broiler chickens. J Anim Sci. 2020;98(4):skaa092. 10.1093/jas/skaa092 10.1093/jas/skaa092 PMC7183351 32207523

[16] Bun SD, Guo YM, Guo FC, Ji FJ, Cao H (2011). Influence of organic zinc supplementation on the antioxidant status and immune responses of broilers challenged with *Eimeria tenella*. Poult Sci.

[17] Ding W, Smulan LJ, Hou NS, Taubert S, Watts JL, Walker AK (2015). s-Adenosylmethionine levels govern innate immunity through distinct methylation-dependent pathways. Cell Metab.

[18] Grimble RF (2006). The effects of sulfur amino acid intake on immune function in humans. J Nutr.

[19] Castro FLdS, Kim WK (2020). Secondary functions of arginine and sulfur amino acids in poultry health: Review. Animals.

[20] Teng P-Y, Liu G, Choi J, Yadav S, Wei F, Kim WK (2023). Effects of levels of methionine supplementations in forms of L- or DL-methionine on the performance, intestinal development, immune response, and antioxidant system in broilers challenged with *Eimeria* spp. Poult Sci.

[21] Liu G, Sharma MK, Tompkins YH, Teng PY, Kim WK (2024). Impacts of varying methionine to cysteine supplementation ratios on growth performance, oxidative status, intestinal health, and gene expression of immune response and methionine metabolism in broilers under *Eimeria* spp. challenge. Poult Sci.

[22] Kitada M, Xu J, Ogura Y, Monno I, Koya D (2020). Mechanism of activation of mechanistic target of rapamycin complex 1 by methionine. Front Cell Dev Biol.

[23] Zhong C, Tong DQ, Zhang YR, Wang XQ, Yan HC, Tan HZ (2022). DL)-methionine and (DL)-methionyl-(DL)-methionine increase intestinal development and activate Wnt/β-catenin signaling activity in domestic pigeons (Columba livia. Poult Sci.

[24] Sampson LL, Davis AK, Grogg MW, Zheng Y (2016). mTOR disruption causes intestinal epithelial cell defects and intestinal atrophy postinjury in mice. FASEB J.

[25] Liu G, Kim WK (2023). The functional roles of methionine and arginine in intestinal and bone health of poultry: Review. Animals.

[26] Atmaca G (2004). Antioxidant effects of sulfur-containing amino acids. Yonsei Med J.

[27] Bin P, Huang R, Zhou X (2017). Oxidation resistance of the sulfur amino acids: methionine and cysteine. Biomed Res Int.

[28] Brosnan JT, Brosnan ME, Bertolo RFP, Brunton JA (2007). Methionine: A metabolically unique amino acid. Livest Sci.

[29] Castro FLS, Tompkins YH, Pazdro R, Kim WK (2020). The effects of total sulfur amino acids on the intestinal health status of broilers challenged with *Eimeria* spp. Poult Sci.

[30] Klein Geltink RI, Pearce EL (2019). The importance of methionine metabolism. eLife.

[31] Sinclair LV, Howden AJM, Brenes A, Spinelli L, Hukelmann JL, Macintyre AN (2019). Antigen receptor control of methionine metabolism in T cells. eLife.

[32] Yun CH, Lillehoj HS, Lillehoj EP (2000). Intestinal immune responses to coccidiosis. Dev Comp Immunol.

[33] Mirzaaghatabar F, Saki AA, Zamani P, Aliarabi H, Hemati Matin HR (2011). Effect of different levels of diet methionine and metabolisable energy on broiler performance and immune system. Food Agr Immunol.

[34] Sigolo S, Deldar E, Seidavi A, Bouyeh M, Gallo A, Prandini A (2019). Effects of dietary surpluses of methionine and lysine on growth performance, blood serum parameters, immune responses, and carcass traits of broilers. J Appl Anim Res.

[35] Wu B, Cui H, Peng X, Fang J, Cui W, Liu X (2013). Pathology of bursae of Fabricius in methionine-deficient broiler chickens. Nutrients.

[36] Toue S, Kodama R, Amao M, Kawamata Y, Kimura T, Sakai R. Screening of toxicity biomarkers for methionine excess in rats. J Nutr. 2006;136(6 Suppl):1716S–21S. 10.1093/jn/136.6.1716S. 10.1093/jn/136.6.1716S 16702345

[37] Krebs HA, Hems R, Tyler B (1976). The regulation of folate and methionine metabolism. Biochem J.

[38] Teng P-Y, Choi J, Yadav S, Tompkins YH, Kim WK (2021). Effects of low-crude protein diets supplemented with arginine, glutamine, threonine, and methionine on regulating nutrient absorption, intestinal health, and growth performance of *Eimeria*-infected chickens. Poult Sci.

[39] Zaĭonts V, Krylov M, Loskot V, Kirillov A. Biosynthesis of folic acid in *Eimeria tenella* (Coccidia). Parazitologiia. 1978;12(1):3–8. 304554

[40] Noack S, Chapman HD, Selzer PM (2019). Anticoccidial drugs of the livestock industry. Parasitol Res.

[41] Lemme A, Hiller P, Klahsen M, Taube V, Stegemann J, Simon I (2019). Reduction of dietary protein in broiler diets not only reduces n-emissions but is also accompanied by several further benefits. J Appl Poult Res.

[42] Wang Y, Zhou J, Wang G, Cai S, Zeng X, Qiao S (2018). Advances in low-protein diets for swine. J Anim Sci Biotechnol.

[43] Liu SY, Macelline SP, Chrystal PV, Selle PH (2021). Progress towards reduced-crude protein diets for broiler chickens and sustainable chicken-meat production. J Anim Sci Biotechnol.

[44] van Harn J, Dijkslag MA, van Krimpen MM (2019). Effect of low protein diets supplemented with free amino acids on growth performance, slaughter yield, litter quality, and footpad lesions of male broilers. Poult Sci.

[45] Attia YA, Bovera F, Wang J, Al-Harthi MA, Kim WK. Multiple amino acid supplementations to low-protein diets: Effect on performance, carcass yield, meat quality and nitrogen excretion of finishing broilers under hot climate conditions. Animals. 2020;10(6):973. 10.3390/ani10060973. 10.3390/ani10060973 PMC7341316 32503244

[46] Cobb500 Broiler Performance & Nutrition Supplement. Cobb-Vantress. 2018. https://www.cobb-vantress.com/assets/5a88f2e793/Broiler-Performance-Nutrition-Supplement.pdf. Accessed 31 Dec 2023.

[47] Cobb Broiler Management Guide. Cobb-Vantress. 2018. https://www.cobb-vantress.com/assets/Cobb-Files/045bdc8f45/Broiler-Guide-2021-min.pdf. Accessed 31 Dec 2023.

[48] Liu J, Teng P-Y, Kim WK, Applegate TJ (2021). Assay considerations for fluorescein isothiocyanate-dextran (FITC-d): an indicator of intestinal permeability in broiler chickens. Poult Sci.

[49] Choi J, Tompkins YH, Teng P-Y, Gogal RM, Kim WK (2022). Effects of tannic acid supplementation on growth performance, oocyst shedding, and gut health of in broilers infected with *Eimeria maxima*. Animals.

[50] Conway DP, McKenzie ME. Poultry coccidiosis: diagnostic and testing procedures. 3rd ed. Ames: Blackwell Pub; 2007.

[51] Castro FLS, Teng PY, Yadav S, Gould RL, Craig S, Pazdro R (2020). The effects of L-Arginine supplementation on growth performance and intestinal health of broiler chickens challenged with *Eimeria* spp. Poult Sci.

[52] Livak KJ, Schmittgen TD (2001). Analysis of relative gene expression data using real-time quantitative PCR and the 2− ΔΔCT method. Methods.

[53] McDougald LR (1998). Intestinal protozoa important to poultry. Poult Sci.

[54] Taylor J, Sakkas P, Kyriazakis I (2022). Starving for nutrients: anorexia during infection with parasites in broilers is affected by diet composition. Poult Sci..

[55] Macelline SP, Chrystal PV, McQuade LR, McLnerney BV, Kim Y, Bao Y (2022). Graded methionine dietary inclusions influence growth performance and apparent ileal amino acid digestibility coefficients and disappearance rates in broiler chickens. Anim Nutr.

[56] Rehman AU, Arif M, Husnain MM, Alagawany M, Abd El-Hack ME, Taha AE, et al. Growth performance of broilers as influenced by different levels and sources of methionine plus cysteine. Animals. 2019;9(12):56. 10.3390/ani9121056. 10.3390/ani9121056 PMC6941102 31805723

[57] Song B, Fu M, He F, Zhao H, Wang Y, Nie Q (2021). Methionine deficiency affects liver and kidney health, oxidative stress, and ileum mucosal immunity in broilers. Front Vet Sci.

[58] Fang CC, Feng L, Jiang WD, Wu P, Liu Y, Kuang SY (2021). Effects of dietary methionine on growth performance, muscle nutritive deposition, muscle fibre growth and type I collagen synthesis of on-growing grass carp (*Ctenopharyngodon idella*). Br J Nutr.

[59] Barekatain R, Nattrass G, Tilbrook AJ, Chousalkar K, Gilani S (2019). Reduced protein diet and amino acid concentration alter intestinal barrier function and performance of broiler chickens with or without synthetic glucocorticoid. Poult Sci.

[60] Lambert W, Berrocoso JD, Swart B, van Tol M, Bruininx E, Willems E (2023). Reducing dietary crude protein in broiler diets positively affects litter quality without compromising growth performance whereas a reduction in dietary electrolyte balance further improves litter quality but worsens feed efficiency. Anim Feed Sci Technol.

[61] Britton W, Hill C, Barber C (1964). A mechanism of interaction between dietary protein levels and coccidiosis in chicks. J Nutr.

[62] Chapman H (1978). Studies on the excystation of different species of *Eimeria* in vitro. Z Parasitenkd.

[63] Tsirtsikos P, Fegeros K, Kominakis A, Balaskas C, Mountzouris KC (2012). Modulation of intestinal mucin composition and mucosal morphology by dietary phytogenic inclusion level in broilers. Animal.

[64] Guo S, Liu L, Lei J, Qu X, He C, Tang S (2021). Modulation of intestinal morphology and microbiota by dietary Macleaya cordata extract supplementation in Xuefeng Black-boned Chicken. Animal..

[65] Sen S, Ingale S, Kim Y, Kim J, Kim K, Lohakare J (2012). Effect of supplementation of *Bacillus subtilis* LS 1–2 to broiler diets on growth performance, nutrient retention, caecal microbiology and small intestinal morphology. Res Vet Sci.

[66] Yu D, Zhu W, Hang S (2019). Effects of long-term dietary protein restriction on intestinal morphology, digestive enzymes, gut hormones, and colonic microbiota in pigs. Animals.

[67] Stover PJ (2004). Physiology of folate and vitamin B12 in health and disease. Nutr Rev.

[68] Kachungwa Lugata J, Ortega ADSV, Szabó C (2022). The role of methionine supplementation on oxidative stress and antioxidant status of poultry-A review. Agriculture.

[69] Fagundes NS, Milfort MC, Williams SM, Da Costa MJ, Fuller AL, Menten JF (2020). Dietary methionine level alters growth, digestibility, and gene expression of amino acid transporters in meat-type chickens. Poult Sci.

[70] Kim WK, Singh AK, Wang J, Applegate T (2022). Functional role of branched chain amino acids in poultry: a review. Poult Sci.

[71] Li P, Yin Y-L, Li D, Woo Kim S, Wu G (2007). Amino acids and immune function. Br J Nutr.

[72] Wu G (2010). Functional amino acids in growth, reproduction, and health. Adv Nutr.

[73] Ross D (1988). Glutathione, free radicals and chemotherapeutic agents. Mechanisms of free-radical induced toxicity and glutathione-dependent protection. Pharmacol Ther.

[74] Pizzorno J (2014). Glutathione!. Integr Med (Encinitas).

[75] Séité S, Mourier A, Camougrand N, Salin B, Figueiredo-Silva AC, Fontagné-Dicharry S (2018). Dietary methionine deficiency affects oxidative status, mitochondrial integrity and mitophagy in the liver of rainbow trout (*Oncorhynchus mykiss*). Sci Rep.

[76] Wu P, Tang L, Jiang W, Hu K, Liu Y, Jiang J (2017). The relationship between dietary methionine and growth, digestion, absorption, and antioxidant status in intestinal and hepatopancreatic tissues of sub-adult grass carp (Ctenopharyngodon idella). J Anim Sci Biotechnol.

[77] Teng PY, Choi J, Yadav S, Marshall B, Castro FLS, Ferrel J (2023). Evaluation of a dacitic (rhyolitic) tuff breccia use on performance, inflammatory, and antioxidant responses in broilers mildly challenged with *Eimeria* spp. Poult Sci.

[78] Rijpert-Duvivier ACM, Geurts CPH, Vangroenweghe F, Allais L, van Doorn DCK (2021). Oocyst shedding patterns of *Eimeria* species and their association with management and performance at ten rose veal starter farms in the Netherlands. Vet Parasitol Reg Stud Reports.

[79] Cha JO, Zhao J, Yang MS, Kim WI, Cho HS, Lim CW (2018). Oocyst-shedding patterns of three *Eimeria* species in chickens and shedding pattern variation depending on the storage period of *Eimeria tenella* oocysts. J Parasitol.

[80] Guijarro MV, Nawab A, Dib P, Burkett S, Luo X, Feely M (2023). TYMS promotes genomic instability and tumor progression in Ink4a/Arf null background. Oncogene.

[81] Burdelski C, Strauss C, Tsourlakis MC, Kluth M, Hube-Magg C, Melling N (2015). Overexpression of thymidylate synthase (TYMS) is associated with aggressive tumor features and early PSA recurrence in prostate cancer. Oncotarget.

